# Comparative metabolic and lipidomic profiling of human breast cancer cells with different metastatic potentials

**DOI:** 10.18632/oncotarget.11560

**Published:** 2016-08-24

**Authors:** Hye-Youn Kim, Kyung-Min Lee, So-Hyun Kim, Yeo-Jung Kwon, Young-Jin Chun, Hyung-Kyoon Choi

**Affiliations:** ^1^ College of Pharmacy, Chung-Ang University, Seoul 156-756, Republic of Korea

**Keywords:** metastasis, breast cancer cells, metabolomics, lipidomics, PLSR

## Abstract

This study conducted comprehensive and comparative metabolic and lipidomic profiling of a human epithelial breast cell line (MCF-10A), a slightly metastatic (MCF-7), and a highly metastatic (MDA-MB-231) breast cancer cell line using gas chromatography mass spectrometry (GC-MS) and direct infusion mass spectrometry (DI-MS). Among 39 metabolites identified by GC-MS analysis, xanthine, glucose-6-phosphate, mannose-6-phosphate, guanine, and adenine were selected as prognostic markers of breast cancer metastasis. Major metabolic pathways involved in differentiation of the cell lines were alanine, aspartate, and glutamate metabolism, purine metabolism and glycine, serine, and threonine metabolism. Among 44 intact lipid species identified by DI-MS analysis, the levels of most phospholipids were higher in both metastatic groups than in normal cells. Specifically, the levels of phosphatidylserine (PS) 18:0/20:4, phosphatidylinositol (PI) 18:0/20:4, and phosphatidylcholine (PC) 18:0/20:4 were markedly higher while those of phosphatidylethanolamine (PE) 18:1/18:1 and PI 18:0/18:1 were lower in MDA-MB-231 cells than in MCF-7 cells. A partial-least-squares regression model was developed and validated for predicting the metastatic potential of breast cancer cells. The information obtained in this study will be useful when developing diagnostic tools and for identifying potential therapeutic targets for metastatic breast cancer.

## INTRODUCTION

Breast cancer is the most common type of cancer in women and the second most common type of cancer overall worldwide. In Europe, Belgium has the highest prevalence of breast cancer, approximately 112 per 100,000 population (World Cancer Research Fund International statistics available at http://www.wcrf.org/int/cancer-facts-figures/data-specific-cancers/breast-cancer-statistics). Among Asian countries, the incidence has increased rapidly in recent years. Especially, in South Korea and Japan, the incidence rates of breast cancer have increased by 6% annually [1]. Adjuvant chemotherapy is offered to most breast cancer patients, but about 40% of early-stage breast cancer patients are at risk of developing distant metastases, which can result in death [2].

Biological markers are needed to identify patients with developing metastases and to improve the clinical management of the disease. Potential biomarkers have been investigated for predicting breast cancer metastases, with some showing clinical efficacy. The developed markers that have true prognostic use for patients with breast cancer include the level of urokinase-type plasminogen activator/plasminogen activator inhibitor 1 protein (uPA/PAI1), steroid-receptor expression, and epidermal growth factor receptor 2 (ERBB2) gene amplification and protein expression [3]. Overexpression of enzymes that degrade the extracellular matrix, such as uPA and matrix metalloproteinases, and oncoproteins such as ERBB2, ras, and c-Myc are known to be associated with a high risk of metastasis [3, 4]. Steroid receptor coactivator-1 expression is known to promote metastasis and is inversely correlated with estrogen receptor (ER) β expression, which is a predictor of a better prognosis in breast cancer [4].

Metabolic profiling has been used to study comprehensive responses when characterizing tumor types in clinical research. Cancer cell analyses using metabolomic techniques have been reported using mass spectrometry for ovarian cancer and other gastrointestinal cancers such as pancreatic cancer and colorectal cancer [5–7]. The construction of a metabolic map of a large cohort of breast cancer tissues and plasma using gas chromatography mass spectrometry (GC-MS), and comparative lipid profiling between breast cancer and normal tissues from humans using ultra-performance liquid chromatography mass spectrometry (UPLC-MS) have been reported [8–10]. In addition, lipidomic analyses of mouse breast cancer cell lines [11] and human breast ductal carcinoma (T47-D) and adenocarcinoma (MDA-MB-231) cell lines have been carried out using thin-layer-chromatography mass spectrometry (TLC-MS) [12].

Two human breast cancer cell lines, MCF-7 and MDA-MB-231, are known to present with different metastasis and metastatic properties and ER positivity in human breast cancers. ER^+^ MCF-7 cells are highly hormone-dependent for growth and have a comparatively low capacity for metastasis among breast tumor cells, while ER^−^ MDA-MB-231 cells are completely hormone-independent and exhibit a high metastatic potential [13, 14]. Previous studies have used these cell lines to identify and assess alterations in genes and proteins associated with metastasis in breast cancer using various types of genomic and proteomic analyses. Of 12,625 genes, 26 were increased in both MCF-7 and MDA-MB-231 cells by hypoxia [13], and the expression of miR-125 was lower in MCF-7 cells but higher in MDA-MB-231 cells than in MCF-10A cells [14]. The level of 14-3-3σ protein was strongly decreased in MCF-7 and MDA-MB-231 cells and in primary breast carcinomas compared to normal cells [15]. However, no previous studies have performed comparative metabolomic and lipidomic profiling of breast cancer cell lines (MCF-7 and MDA-MB-231) with different degrees of metastasis.

In this study, we performed comprehensive and comparative metabolomic and lipidomic profiling of breast cancer cell lines with different degrees of metastasis (MCF-7 and MDA-MB-231 cells) using GC-MS and direct infusion mass spectrometry (DI-MS) for the first time. In particular, DI-MS analysis using nano-electrospray mass spectrometry facilitated the analysis of intact lipid species in a rapid, simple, and highly sensitive manner. In addition, a predictive model for the metastasis stage of breast cancer was developed using the metabolic and lipidomic data sets obtained in this study by partial-least-squares discriminant analysis (PLS-DA) and partial-least-squares (PLS) projection to latent structures regression.

## RESULTS

### Comparative metabolic profiling of breast cancer cells with different metastatic potentials using GC-MS

Comprehensive metabolic profiling of human mammary epithelial and breast cancer cell extracts was performed using GC-MS to investigate the profiles of major compounds that are detected in breast cancer cells according to different metastasis stages. As listed in Table 1, the following 39 metabolites were identified in human mammary epithelial and breast cancer cells: 2 alcohols (erythritol and myo-inositol), 14 amino acids (alanine, asparagine, aspartic acid, glutamine, glycine, leucine, lysine, ornithine, proline, serine, threonine, tryptophan, tyrosine, and valine), 4 fatty acids (3-hydroxybutanoic acid, oleic acid, palmitic acid, and stearic acid), 5 organic acids (isocitric acid, lactic acid, malic acid, oxalic acid, and pyruvic acid), 6 purines (adenine, guanine, hypoxanthine, inosine, uracil, and xanthine), 7 sugars (6-phosphogluconic acid, fructose-6-phosphate, glucose, glucose-6-phosphate, glyceric acid, mannose-6-phosphate, and sucrose), and creatinine.

**Table 1:** Mass fragment of metabolites contained in breast cancer cells and human mammary epithelial cells

Compound	RT (min)	Fragmentation ion (m/z)	TMS^a^
**Alcohol**			
Erythritol	19.03	103, 129, 205, **217**	4
	19.05		
Myo-inositol	28.95	191, 217, 265, **305**	6
**Amino acid**			
Alanine	7.00	**116**, 133, 190, 218	2
Asparagine	20.85	**116**, 132, 188, 231	3
Aspartic acid	15.06	117, 130, **160**, 245	2
	17.41		
	17.43		
Glutamine	19.78	128, 230, **246**, 348	3
	19.79		
	19.81		
	23.11	**156**, 203, 246, 347	3
Glycine	7.47	86, **102**, 176, 204	2
	12.02	86, **174**, 248, 276	3
	12.03		
Leucine	8.30	**86**, 103, 170, 188	1
	11.72	**158**, 218, 232, 260	2
Lysine	26.08	156, **174**, 230, 317	4
Ornithine	24.00	100, **142**, 200, 420	4
Proline	11.81	133, **142**, 216, 230	2
Serine	10.81	103, 116, **132**, 219	2
	13.45	100, **204**, 218, 278	3
Threonine	11.74	**117**, 130, 158, 219	2
	11.75		
	14.08	117, **218**, 291, 320	3
	14.09		
Tryptophan	30.92	**202**, 218, 291, 405	3
Tyrosine	26.37	179, **218**, 280, 382	3
Valine	6.71	**72**, 130, 156, 174	1
	9.74	100, **144**, 174, 218	2
	9.76		
**Fatty acid**			
3-hydroxybutanoic acid	8.05	117, 130, **191**, 204	2
Oleic acid	31.08	**117**, 129, 145, 339	1
Palmitic acid	28.36	117, 129, 145, **313**	1
Stearic acid	31.49	**117**, 129, 145, 341	1
**Organic acid**			
Isocitric acid	24.07	**273**, 305, 347, 465	4
Lactic acid	6.04	**117**, 133, 191, 219	2
Malic acid	16.67	189, **233**, 245, 335	3
Oxalic acid	7.90	100, 117, **133**, 190	2
Pyruvic acid	7.63	89, 131, 189, **218**	2
**Purine**			
Adenine	24.95	**116**, 133, 190, 218	2
Guanine	29.59	171, 264, **352**, 367	3
Hypoxanthine	23.73	193, 206, **265**, 280	2
Inosine	36.11	193, **217**, 245, 281	4
Uracil	12.86	**99**, 113, 241, 256	2
Xanthine	27.80	279, 294, **353**, 368	3
**Sugar**			
6-phosphogluconic acid	33.93	217, **299**, 357, 387	7
Fructose-6-phosphate	32.31	217, **315**, 357, 387	6
Glucose	27.15	129, 191, **204**, 217	5
Glucose-6-phosphate	32.32	129, 204, 299, **387**	6
	32.49		
	32.74		
	33.03		
	33.33		
	33.50		
Glyceric acid	12.67		
Mannose-6-phosphate	35.72	217, 299, 357, **387**	6
Sucrose	36.96	217, 271, **361**, 437	8
**Other**			
Creatinine	18.10	100, **115**, 143, 329	3

a TMS = trimethylsilylation.

**Figure 1:**
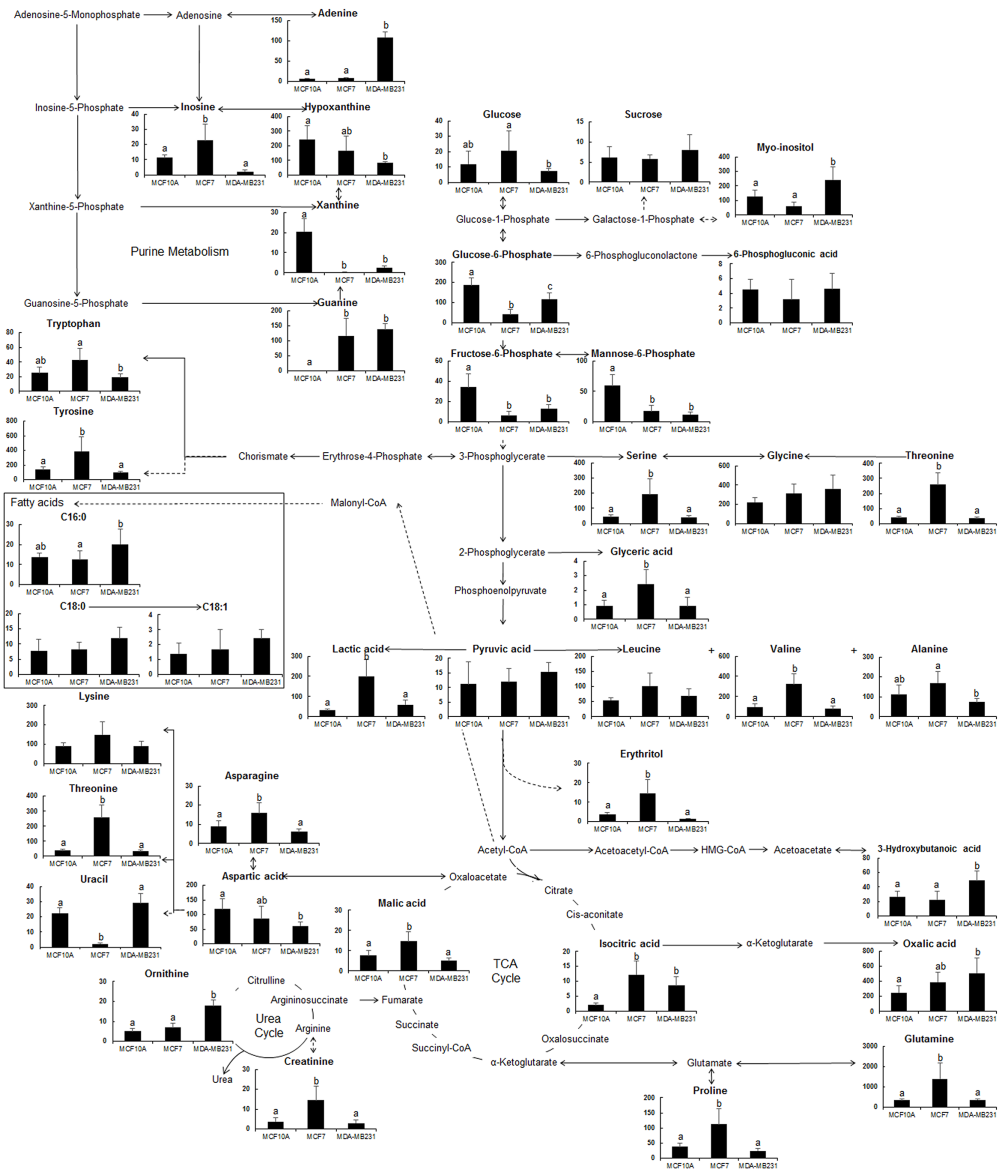
Schematic diagram of the metabolic pathway and relative levels of detected compounds in breast cancer cells with different metastatic potentials Schematic diagram modified from the pathway presented in the KEGG database (http://www.genome.jp/kegg/). ANOVA was carried out to detect statistically significant differences between the samples (*p* <0.05). Data are presented as mean values with error bars representing standard deviation (*n* = 5). Different letters indicate statistically significant differences between metabolite levels.

Figure 1 depicts the interconnecting cellular metabolic pathways with superimposed levels of metabolites found in human breast cells with varying degrees of metastasis; the corresponding data are provided in Supplementary Table S1. ANOVA was performed to assess the statistical significance of the relative levels of each metabolite among different samples (*p <* 0.05). The levels of one alcohol (erythritol), amino acids (asparagine, glutamine, proline, serine, threonine, tyrosine, and valine), organic acids (isocitric acid, lactic acid, and malic acid), one purine (guanine), one sugar (glyceric acid), and creatinine were significantly higher in slightly metastatic cancer cells (MCF-7) than in normal breast cells (MCF-10A) and highly metastatic cells (MDA-MB-231). In contrast, the levels of purines such as uracil and xanthine and sugars such as fructose-6-phosphate, glucose-6-phosphate, and mannose-6-phosphate were significantly lower in MCF-7 cells than in MCF-10A and MDA-MB-231 cells.

In highly metastatic cells (MDA-MB-231), the levels of alcohol (including erythritol), amino acids (including asparagine, glutamine, proline, serine, threonine, tyrosine, and valine), organic acids (including lactic acid and malic acid), purine (including inosine), and creatinine were reduced. In contrast, the level of uracil was higher than that in normal cells (MCF-10A). Isocitric acid and guanine levels remained high, whereas xanthine, fructose-6-phosphate, and mannose-6-phosphate levels stayed low. The levels of myo-inositol, ornithine, 3-hydoxybutanoic acid, oxalic acid, and adenine were significantly higher while the levels of hypoxanthine were significantly lower in MDA-MB-231 cells than in MCF-7 cells. The glucose-6-phosphate level was also higher in MDA-MB-231 cells than in MCF-7 cells, but was not as high as that in MCF-10A cells.

Major pathways involved in the metabolic networks of human mammary epithelial and breast cancer cells were determined by annotating the identified metabolites using the MetaboAnalyst platform (http://www.metaboanalyst.ca). A list of 39 identified metabolites was used as an input for pathway analysis, and *Homo sapiens* (human; 80 pathways) was selected for further enrichment analyses. The most relevant pathways and metabolites that were strongly represented in metastatic breast cancer cells are listed in Table 2. These metabolic pathways were selected using the impact score and –log(*p*) value. The impact score was determined by the pathway topological importance of the metabolites, and –log(*p*) was used as the enrichment score, reflecting the probability of the pathway being identified at random; the number of ‘Hits’ was the actual number of matched metabolites in the pathway [16]. The following 10 metabolites were associated with aminoacyl-tRNA biosynthesis (*p* = 0.000): asparagine, leucine, lysine, glutamine, glycine, proline, serine, threonine, tyrosine, and valine. The following seven metabolites were associated with purine metabolism (*p* = 0.001): adenine, glutamine, glycine, guanine, hypoxanthine, inosine, and xanthine. In addition, five metabolites were associated with glycine, serine, and threonine metabolism (glyceric acid, glycine, pyruvic acid, serine, and threonine; *p* = 0.001) and with arginine and proline metabolism (creatinine, glutamine, ornithine, proline, and pyruvic acid; *p* = 0.007). Moreover, alanine, aspartate, and glutamate metabolism; glyoxylate and dicarboxylate metabolism; the pentose phosphate pathway; glycolysis or gluconeogenesis; starch and sucrose metabolism; and the tricarboxylic acid cycle were all associated with the metastasis of breast cancer cells. These various metabolic networks represent potential therapeutic targets that could be utilized in the development of new anticancer drugs for breast cancer.

**Table 2:** List of selected pathways identified by pathway analysis using MetaboAnalyst

No.	Interaction metabolite	Pathway name	Total cmpd^a^	Expected	Hitscmpd^b^	Raw *p*cmpd^c^	-log(*p*)	Impactcmpd^d^
1	Glyceric acid, glycine, pyruvic acid, serine, threonine	Glycine, serine and threonine metabolism	48	0.78	5	0.0009	7.0325	0.4209
2	Asparagine, aspartic acid, glutamine, pyruvic acid	Alanine, aspartate and glutamate metabolism	24	0.39	4	0.0005	7.6095	0.2754
3	Creatinine, glutamine, ornithine, proline, pyruvic acid	Arginine and proline metabolism	77	1.25	5	0.0072	4.9304	0.2383
4	Glyceric acid, isocitric acid, pyruvic acid, oxalic acid	Glyoxylate and dicarboxylate metabolism	50	0.81	4	0.0079	4.8364	0.1868
5	6-phosphogluconic acid, glucose, glyceric acid, pyruvic acid	Pentose phosphate pathway	32	0.52	4	0.0015	6.4838	0.1574
6	Isocitric acid, pyruvic acid	Citrate cycle (TCA cycle)	20	0.32	2	0.0404	3.2077	0.1485
7	Asparagine, leucine, lysine, glutamine, glycine, proline, serine, threonine, tyrosine, valine	Aminoacyl-tRNA biosynthesis	75	1.22	10	0.0000	15.75	0.1127
8	Glucose, lactic acid, pyruvic acid	Glycolysis or Gluconeogenesis	31	0.50	3	0.0129	4.3487	0.0953
9	Glucose, glucose-6-phosphate, sucrose	Starch and sucrose metabolism	50	0.81	3	0.0456	3.0869	0.0768
10	Adenine, glutamine, glycine, guanine, hypoxanthine, inosine, xanthine	Purine metabolism	92	1.49	7	0.0005	7.5315	0.0636

a Total cmpd is the total number of compounds in the pathway.

b Hits is the actual matched number from the uploaded data.

c Raw *p* is the original *p*-value calculated from the pathway analysis.

d Impact is the pathway impact value calculated from pathway topology analysis.

### Comparative lipidomic profiling of breast cancer cells with different metastatic potentials using DI-MS

The nonpolar phase of extracts from human mammary epithelial and breast cancer cells, was used to investigate the lipid profile. Representative lipid spectra of samples derived from a pooled extract and MS/MS spectra of the assigned 44 intact lipids are presented in Supplementary Figure S1 and S2. In total, 10 phosphatidylcholine (PC) species and one each of plasmenylphosphatidylcholine (plasmenyl-PC), plasmenylphosphatidylethanolamine (plasmenyl-PE), and sphingomyelin (SM) species were detected in positive ion mode, while four phosphatidylethanolamine (PE), one plasmenyl-PE, fifteen phosphatidylserine (PS), nine phosphatidylinositol (PI), and two phosphatidylglycerol (PG) species were detected in negative ion mode. In addition to identifying lipid species in the three different cell types, the relative abundance of each lipid in relation to the metastatic potential is listed in Supplementary Table S2. ANOVA with a Tukey's *post hoc* test revealed significant differences between the cell groups (*p <* 0.05).

Table 3 lists the relative changes in lipid species between MCF-7 and MCF-10A cells, MDA-MB-231 and MCF-10A cells, and MDA-MB-231 and MCF-7 cells, with *p* values determined using Student's *t*-tests. The relative changes between cell types ranged from 0.10- to 17.01-fold. All PCs, PEs, and PGs showed relative changes of >1.0-fold in MCF-7 cells, most or all PCs, PEs, PSs, and PIs also showed relative changes of >1.0-fold in MDA-MB-231 cells relative to the corresponding levels found in MCF-10A cells. In addition, compared to MDA-MB-231 and MCF-7, the relative changes in plasmenyl-PEs and most of the identified PSs and PIs were greater than 1.0-fold (which indicates higher levels in MDA-MB-231 than in MCF-7 cells), whereas those of plasmenyl-PC, PEs, and PGs were less than 1.0-fold (which indicates lower levels in MDA-MB-231 than in MCF-7 cells), and most PCs and SM levels did not change significantly.

**Table 3:** Identification of lipid species from human MCF-10A mammary epithelial cells and MCF-7 and MDA-MB-231 human breast cancer cells by nano-electrospray mass spectrometry, along with *p* values and relative changes in identified peak intensities

Ion mode	Ion species	m/z	Proposed composition	MCF-7/MCF-10A		MDA-MB-231/ MCF-10A		MDA-MB-231/MCF-7	
				*p*	Fold change	*p*	Fold change	*p*	Fold change
				PC					
(+)	[M + H]^+^	758	C16:1/C18:1	0.000	1.99 (↑)	0.000	2.47 (↑)	0.136	1.24 (ns)
(+)	[M + H]^+^	784	C18:1/C18:2	0.000	2.40 (↑)	0.004	4.10 (↑)	0.063	1.71 (ns)
(+)	[M + H]^+^	786	C18:1/C18:1	0.000	2.54 (↑)	0.002	4.07 (↑)	0.064	1.60 (ns)
(+)	[M + H]^+^	798	C18:3/C19:0	0.287	1.43 (ns)	0.636	1.16 (ns)	0.240	0.81 (ns)
(+)	[M + H]^+^	808	C18:2/C20:3	0.000	3.19 (↑)	0.005	4.48 (↑)	0.223	1.40 (ns)
(+)	[M + H]^+^	810	C18:0/C20:4	0.004	1.59 (↑)	0.000	4.48 (↑)	0.000	2.81 (↑)
(+)	[M + H]^+^	814	C18:1/C20:1	0.000	1.87 (↑)	0.829	1.02 (ns)	0.000	0.55 (↓)
(+)	[M + H]^+^	816	C18:1/C20:0	0.001	1.57 (↑)	0.000	1.83 (↑)	0.145	1.16 (ns)
(+)	[M + H]^+^	818	C18:0/C20:0	0.192	1.51 (ns)	0.017	2.28 (↑)	0.134	1.51 (ns)
(+)	[M + H]^+^	834	C18:1/C22:5	0.000	1.57 (↑)	0.000	1.52 (↑)	0.634	0.97 (ns)
plasmenyl-PC									
(+)	[M + H]^+^	800	C20:0/C18:1	0.000	1.93 (↑)	0.859	0.95 (ns)	0.004	0.49 (↓)
plasmenyl-PE									
(+)	[M + H]^+^	704	C18:0/C16:0	0.108	1.49 (ns)	0.028	1.53 (↑)	0.886	1.02 (ns)
SM									
(+)	[M]^+^	703	D18:1/16:0	0.750	1.09 (ns)	0.094	1.50 (ns)	0.157	1.37 (ns)
PE									
(−)	[M − H]^−^	742	C18:1/C18:1	0.000	1.62 (↑)	0.000	0.47 (↓)	0.000	0.29 (↓)
(−)	[M − H]^−^	746	C17:0/C19:0	0.000	2.62 (↑)	0.047	1.56 (↑)	0.002	0.60 (↓)
(−)	[M − H]^−^	764	C18:1/C20:4	0.000	2.68 (↑)	0.035	1.23 (↑)	0.000	0.46 (↓)
(−)	[M − H]^−^	792	C18:1/C22:4	0.000	1.67 (↑)	0.002	1.55 (↑)	0.350	0.93 (ns)
plasmenyl-PE									
(−)	[M − H]^−^	750	C18:0/C20:4	0.000	0.36 (↓)	0.000	2.87 (↑)	0.000	7.90 (↑)
PS									
(−)	[M − H]^−^	758	C16:1/C18:1	0.000	1.98 (↑)	0.000	3.32 (↑)	0.001	1.67 (↑)
(−)	[M − H]^−^	760	C16:0/C18:1	0.000	1.48 (↑)	0.000	2.44 (↑)	0.000	1.65 (↑)
(−)	[M − H]^−^	762	C16:0/C18:0	0.006	1.22 (↑)	0.000	2.06 (↑)	0.000	1.70 (↑)
(−)	[M − H]^−^	774	C17:0/C18:1	0.000	2.46 (↑)	0.025	1.21 (↑)	0.000	0.49 (↓)
(−)	[M − H]^−^	784	C18:1/C18:2	0.000	0.60 (↓)	0.000	3.29 (↑)	0.000	5.45 (↑)
(−)	[M − H]^−^	786	C18:0/C18:2	0.796	0.99 (ns)	0.000	2.12 (↑)	0.000	2.15 (↑)
(−)	[M − H]^−^	788	C18:0/C18:1	0.007	0.86 (↓)	0.000	2.04 (↑)	0.000	2.37 (↑)
(−)	[M − H]^−^	790	C18:0/C18:0	0.000	2.01 (↑)	0.000	2.11 (↑)	0.241	1.05 (ns)
(−)	[M − H]^−^	810	C18:0/C20:4	0.468	1.07 (ns)	0.000	4.43 (↑)	0.000	4.15 (↑)
(−)	[M − H]^−^	816	C18:1/C20:0	0.000	1.57 (↑)	0.000	1.40 (↑)	0.031	0.90 (↓)
(−)	[M − H]^−^	834	C18:0/C22:6	0.000	1.19 (↑)	0.000	4.17 (↑)	0.000	2.15 (↑)
(−)	[M − H]^−^	836	C18:1/C22:4	0.000	2.45 (↑)	0.000	2.41 (↑)	0.716	0.98 (ns)
(−)	[M − H]^−^	838	C18:0/C22:4	0.331	1.07 (ns)	0.000	3.76 (↑)	0.000	3.52 (↑)
(−)	[M − H]^−^	842	C18:1/C22:1	0.000	0.64 (↓)	0.180	1.07 (ns)	0.000	1.67 (↑)
(−)	[M − H]^−^	844	C18:1/C22:0	0.000	1.58 (↑)	0.000	1.37 (↑)	0.111	0.87 (ns)
				PI					
(−)	[M − H]^−^	807	C16:0/C16:1	0.000	3.23 (↑)	0.000	17.01 (↑)	0.000	5.27 (↑)
(−)	[M − H]^−^	833	C16:1/C18:1	0.021	1.14 (↑)	0.000	2.10 (↑)	0.000	1.84 (↑)
(−)	[M − H]^−^	835	C16:0/C18:1	0.000	2.46 (↑)	0.009	1.40 (↑)	0.000	0.57 (↓)
(−)	[M − H]^−^	861	C18:1/C18:1	0.000	0.66 (↓)	0.000	0.25 (↓)	0.000	0.37 (↓)
(−)	[M − H]^−^	863	C18:0/C18:1	0.000	1.67 (↑)	0.000	0.16 (↓)	0.000	0.10 (↓)
(−)	[M − H]^−^	881	C16:0/C22:6	0.056	1.58 (ns)	0.005	3.83 (↑)	0.011	2.43 (↑)
(−)	[M − H]^−^	883	C18:1/C20:4	0.024	1.25 (↑)	0.000	4.23 (↑)	0.000	3.38 (↑)
(−)	[M − H]^−^	885	C18:0/C20:4	0.000	0.69 (↓)	0.000	5.80 (↑)	0.000	8.38 (↑)
(−)	[M − H]^−^	887	C18:0/C20:3	0.000	0.48 (↓)	0.038	1.17 (↑)	0.000	2.42 (↑)
				PG					
(−)	[M − H]^−^	747	C16:0/C18:1	0.000	1.64 (↑)	0.026	0.72 (↓)	0.000	0.44 (↓)
(−)	[M − H]^−^	773	C18:1/C18:1	0.000	2.02 (↑)	0.000	0.22 (↓)	0.000	0.11 (↓)

PC, phosphatidylcholine; plasmenyl-PC, plasmenylphosphatidylcholine; plasmenyl-PE, plasmenylphosphatidylethanolamine; PE, phosphatidylethanolamine; PS, phosphatidylserine; PI, phosphatidylinositol; PG, phosphatidylglycerol.

ns, no significant difference (*p* > 0.05); ↑, significant difference (*p* < 0.05) and relative fold change > 1; ↓, significant difference (*p* < 0.05) and relative fold change < 1.

### Development of a predictive model for breast cancer cells according to the metastatic potential

**Figure 2:**
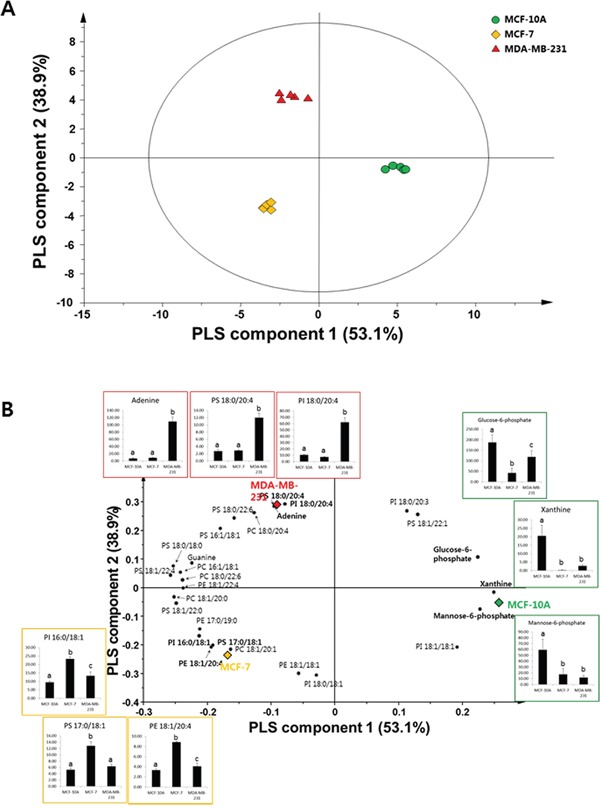
**A.** PLS-DA-derived score plot for mammary epithelial and metastatic breast cancer cells (*n* = 5 for each group). **B.** PLS-DA loading plot of variables with a VIP value of >1.1 explaining the separation above The graphs show changes in major compounds in each group. Data represent mean values with error bars representing the standard deviation values. Different letters on the graph indicate statistically significant differences between samples based on ANOVA with Tukey’*s post hoc* test. ●: MCF-10A; ♦: MCF-7; ▲: MDA-MB-231

To obtain models that enable discrimination of the three different cell types, and identify potential biomarkers for breast cancer, PLS-DA was performed using the total GC-MS and DI-MS data sets from normal cells (MCF-10A), slightly metastatic cells (MCF-7), and highly metastatic cells (MDA-MB-231). The variable influence on projection (VIP) value reflects the influence of components that contribute to separation in the PLS-DA models. Generally, VIP cutoff values of 0.7–0.8 have been accepted for variable selection, with those larger than 1.0 being the most influential for the model [17]. In this study, six PLS-DA models were developed for compounds selected using various VIP cutoff values (0, 0.7, 0.9, 1.0, 1.1, and 1.2) to predict breast cancer metastasis. The optimal PLS-DA model was determined based on the values of *R*^2^*Y* (goodness-of-fit parameter) and *Q*^2^*Y* (predictive ability parameter), with values close to 1 being indicative of a perfect model. The best *R*^2^*Y* value of 0.990 and *Q*^2^*Y* value of 0.984 were obtained when the VIP cutoff value was >1.1 (Table 4). The PLS-DA model validity was investigated by performing 20-times random permutation testing, with the results showing that the model was valid (with an *R*^2^*Y* intercept of 0.019 and a *Q*^2^*Y* intercept of −0.336). In addition, the score plot derived from the optimal PLS-DA model demonstrated that the normal group and two breast cancer groups could be clearly separated by PLS component 1, and that the slightly metastatic and highly metastatic cancer groups could be separated by PLS component 2 (Figure 2A). The corresponding loading plot showed the important variables responsible for the clustering observed in the score plot. From the loading plot, glucose-6-phosphate, xanthine, and mannose-6-phosphate were assigned as key metabolites in MCF-10A cells, and the levels of PI 16:0/18:1, PS 17:0/18:1, and PE 18:1/20:4 were highest in MCF-7 cells. Adenine, PS 18:0/20:4, and PI 18:0/20:4 were identified as the main metabolites in MDA-MB-231 cells responsible for discrimination among groups (Figure 2B).

**Table 4:** PLS-DA model parameters using selected metabolomes and lipidomes for VIP cutoff values of 0, 0.7, 0.9, 1.0, 1.1, and 1.2

VIP cut-off values	Total no. of compounds	Compounds^a^		*R^2^Y*	*Q^2^Y*	*R^2^Y-* intercept	*Q^2^Y-* intercept	Component No.
		GC-MS	DI-MS					
0	83	1-39	1-44	0.982	0.957	0.371	−0.299	3
0.7	75	1-17, 21-24, 26-31, 33-37, 39	1-3, 5-12, 14-44	0.984	0.959	0.355	−0.444	3
0.9	63	1, 2, 4, 6, 10-13, 15, 16, 21-23, 26, 27, 29-31, 33, 35-37, 39	1-3, 5-8, 10, 11, 14-44	0.985	0.962	0.267	−0.292	3
1.0	48	10, 13, 16, 21, 22, 26, 27, 30, 31, 33, 35, 37	1, 3, 6-8, 10, 14-38, 40-44	0.986	0.981	0.053	−0.318	2
1.1	27	26, 27, 31, 35, 37	1, 6-8, 10, 14-17, 19, 22, 26, 27, 29, 30, 32, 33, 36-38, 41, 42	0.990	0.984	0.019	−0.336	2
1.2	4	–	8, 26, 30, 33	0.852	0.776	0.097	−0.331	3

a Compounds corresponding to numbers were listed in Supplementary Table S1 and S2.

**Figure 3:**
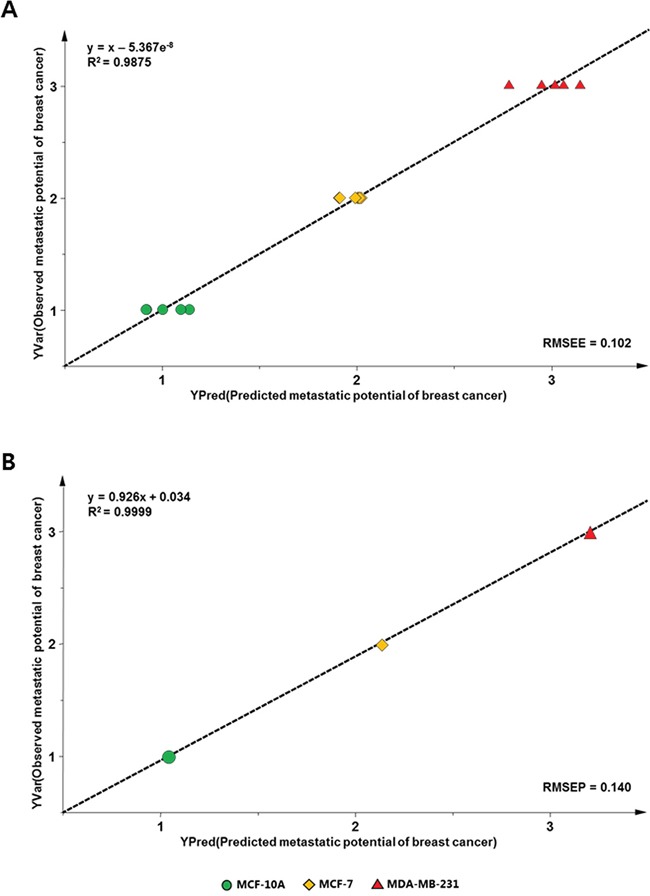
PLS-derived relationship between observed and predicted metastatic potentials of mammary epithelial and metastatic breast cancer cell samples using the training set A. and test set B. analyzed with variables having a VIP value of >1.1

For external validation, a PLS regression model was developed from the training data set of five individual samples of each of MCF-10A, MCF-7, and MDA-MB-231 cells, and contained variables with optimal VIP values. Cross-validation and permutation testing were performed to validate the prediction model, and the differences between the observed and predicted metastatic potentials of breast cancer cells were calculated and expressed as the root-mean-square error of estimation (RMSEE). In addition, one experimental data set from an independent sample was used as the test set and was imported into the PLS calibration plot—constructed by the training set—to yield the root-mean-square error of prediction (RMSEP). The plots and parameters are presented in Figure 3 and Table 5. In this model, the values of RMSEE and RMSEP were calculated to be 0.102 and 0.140, respectively, which indicated the high accuracy of the model. These results indicate that the PLS model used in this study is a potentially useful predictive model for determining the degree of metastasis in cell samples obtained from patients.

**Table 5:** Validation of quality parameters of PLS projection to latent structures regression models derived from the data sets of the metabolomes and lipidomes in mammary epithelial and breast cancer cells

PLS parameters	Values
*R^2^Y*	0.987
*Q^2^Y*	0.981
*R^2^Y* intercept	0.154
*Q^2^Y* intercept	−0.452
RMSEE	0.102
RMSEP	0.140

There were two PLS components

For potential biomarker discovery, metabolites and lipids were selected with VIP values of >1.1 in the PLS-DA model. Among them, the top five ranked lipid species with higher relative changes were selected as effective biomarkers for metastatic breast cancer. The selected biomarkers are listed in Table 6. Five potential biomarkers for metastatic cancer cells were discovered in the GC-MS analysis. Among these biomarkers, the guanine level was 312.9-fold higher (*p =* 0.002) while the levels of xanthine, glucose-6-phosphate, and mannose-6-phosphate were lower in MCF-7 cells than in MCF-10A cells (all *p* values <0.005). The levels of guanine and adenine were higher (all *p* values <0.001) while those of xanthine, glucose-6-phosphate, and mannose-6-phosphate were lower (all *p* values <0.02) in MDA-MB-231 cells than in MCF-10A cells. The levels of xanthine, glucose-6-phosphate, and adenine were higher in MDA-MB-231 cells than in MCF-7 cells (all *p* values <0.03). The levels of potential lipid biomarkers PS 18:1/22:4, PE 18:1/20:4, PI 16:0/18:1, PS 17:0/18:1, and PE 17:0/19:0 were all higher in slightly metastatic MCF-7 cells than in MCF-10A cells, with relative changes greater than 2.45-fold (all *p* values <0.001). The levels of these lipids were higher in MCF-7 cells than in the other two cell types. The levels of PS 18:0/22:6, PS 18:0/20:4, PI 18:0/20:4, and PC 18:0/20:4 were higher while the level of PI 18:0/18:1 was lower in highly metastatic MDA-MB-231 cells than in MCF-10A cells. In particular, the level of PS 18:0/22:6 was highest in MDA-MB-231 cells, followed by MCF-7 and MCF-10A cells. Additionally, the levels of PS 18:0/20:4, PI 18:0/20:4, and PC 18:0/20:4 were markedly higher in the highly metastatic MDA-MB-231 cells than in MCF-7 cells, with relative changes of 4.15-, 8.38-, and 2.81-fold, respectively (all *p* values <0.001). In contrast, the levels of PE 18:1/18:1 and PI 18:0/18:1 were markedly lower in cells with high metastatic potential than in slightly metastatic cells.

**Table 6:** Potential biomarkers of breast cancer metastasis identified by GC-MS and DI-MS, along with *p* values and relative changes in identified peak intensities

MCF-7/MCF-10A				MDA-MB-231/MCF-10A				MDA-MB-231/MCF-7			
Compound	VIP^*a*^	Fold change^*b*^	*p*-value	Compound	VIP^*a*^	Fold change^*b*^	*p*-value	Compound	VIP^*a*^	Fold change^*b*^	*p*-value
**GC-MS**											
Xanthine	1.20	0.02 (↓)	0.000	Xanthine	1.20	0.13 (↓)	0.000	Xanthine	1.20	8.35 (↑)	0.000
Glucose-6-phosphate	1.15	0.22 (↓)	0.000	Glucose-6-phosphate	1.15	0.63 (↓)	0.011	Glucose-6-phosphate	1.15	2.84 (↑)	0.002
Mannose-6-phosphate	1.14	0.29 (↓)	0.002	Mannose-6-phosphate	1.14	0.20 (↓)	0.000	Mannose-6-phosphate	1.14	0.68 (ns)	0.269
Guanine	1.11	312.9 (↑)	0.002	Guanine	1.11	371.5 (↑)	0.000	Guanine	1.11	1.19 (ns)	0.452
Adenine	1.11	1.23 (ns)	0.088	Adenine	1.11	16.26 (↑)	0.000	Adenine	1.11	13.18 (↑)	0.000
**DI-MS**											
PS 18:1/22:4	1.24	2.45 (↑)	0.000	PS 18:0/22:6	1.14	4.17 (↑)	0.000	PS 18:0/20:4	1.11	4.15 (↑)	0.000
PE 18:1/20:4	1.20	2.68 (↑)	0.000	PS 18:0/20:4	1.11	4.43 (↑)	0.000	PE 18:1/18:1	1.11	0.29 (↓)	0.000
PI 16:0/18:1	1.19	2.46 (↑)	0.000	PI 18:0/20:4	1.10	5.80 (↑)	0.000	PI 18:0/20:4	1.10	8.38 (↑)	0.000
PS 17:0/18:1	1.18	2.46 (↑)	0.000	PC 18:0/20:4	1.10	4.48 (↑)	0.000	PC 18:0/20:4	1.10	2.81 (↑)	0.000
PE 17:0/19:0	1.15	2.62 (↑)	0.000	PI 18:0/18:1	1.10	0.16 (↓)	0.000	PI 18:0/18:1	1.10	0.10 (↓)	0.000

a variable influence on projection.

b ↑, significant difference (*p* < 0.05) and relative fold change > 1; ↓, significant difference (*p* < 0.05) and relative fold change < 1.

## DISCUSSION

This study performed cellular metabolic and lipidomic profiling of breast cancer cells with different metastatic potentials. This is the first study to analyze the metabolic profiles of breast cancer cells with different metastatic potentials, and to compare normal controls with two breast cancer cell groups.

The levels of ornithine, adenine, and guanine were significantly higher in highly metastatic cancer cells (MDA-MB-231) than in normal cells (MCF-10A). These metabolites were also found to be significantly elevated in breast cancer tissue compared to normal tissue in a previous study that investigated a large cohort of breast cancer cases [8]. Via the action of ornithine decarboxylase (ODC), ornithine is the starting point for the synthesis of polyamines that are associated with tumor promotion and progression [18]. That study found that the level of ODC expression was highest in MDA-MB-231 cells, which implies that ornithine plays an important role in determining tumor growth and aggressiveness. Increase in adenine has also been found in human gastric cancer cells, and it was speculated that this phenomenon is necessary for ATP transfer and synthesizing nucleic acids for abnormal cell proliferation [19]. Guanine is reportedly the most readily oxidized base in cancer cells, which makes it vulnerable to DNA mutations [20]. Furthermore, adenine and guanine are obtained from inosine monophosphate during purine metabolism. Imbalanced enzymatic activities during purine metabolism, such as a decrease in xanthine oxidase, were found to be associated with the transformation and progression of cancer cells, suggesting that there is a correlation between increase in adenine and guanine and cancer cell metastasis [21]. A metabolomic study using mouse tumor models with different metastatic potentials found that the level of guanine was higher in highly metastatic cell lines (4TO7, 66cl4, and 4T1) than in a slightly metastatic cell line (168FARN) [22]. This implies that there is a relationship between changes in purine metabolism and the metastatic potential of breast cancer cells.

Aminoacyl-tRNA biosynthesis was found to be activated in breast cancer tissues in a previous study of breast cancer and normal tissues [8]. In our study, these amino acids were increased in slightly metastatic cells (MCF-7), but their levels were at control levels in MDA-MB-231 cells with a high metastatic potential. Lower levels in cells with greater metastasis could be due to their greater consumption of amino acids [22]. In particular, among amino acids, glutamine has been known to be a major source of carbon for fatty acid synthesis in cancer cells with defective mitochondria, and these fatty acids can be used for energy storage [23].

The level of PE 18:1/18:1 was higher in MCF-7 cells than in MCF-10A and MDA-MB-231 cells in our study. The level of PE 18:1/18:1 was previously found to be higher in T-47D cells than in MDA-MB-231 cells [12]. In addition, the level of plasmenyl-PE 18:0/20:4 was significantly higher in MDA-MB-231 cells but significantly lower in MCF-7 cells than in MCF-10A cells. These differences may be associated with differences in the hormonal status and metastatic potential between breast cancer cell lines. It was reported that the levels of PE were higher in ER^+^ hormone-sensitive cell lines (MCF-7, LCC2, and MIII) than in ER^−^ hormone-resistant cell lines (MDA-MB-231 and MDA-MB-435), whereas plasmenyl-PE was either absent or present only at a very low level in the ER^+^ cell lines, but was present at a significant level in ER^−^ cell lines [24].

Moreover, it was found for the first time that the levels of PG 16:0/18:1 and PG 18:1/18:1 were higher in MCF-7 cells but lower in MDA-MB-231 cells than in MCF-10A cells. PG is an important precursor of cardiolipin (CL), which plays a critical role in mitochondrial structure and function. PG deficiency results in CL deficiency and it may cause mitochondrial dysfunction in highly metastatic cancer cells [25].

Most phospholipids are more abundant in breast cancer cells than in normal cells. A previous study found similar results in human breast cancer tissues [10]. It was reported that the levels of membrane phospholipids, including PC, PE, PI, SM, and ceramides, were higher in breast cancer tissue than in normal tissue samples, with the levels being maximal in the most aggressive tumors. It has been suggested that overexpression of fatty acid synthase plays an important role in the tumorigenesis, and that *de novo* synthesized fatty acids are required for the rapid proliferation of cancer cells. High levels of lipogenic enzymes and related gene expression were observed in several types of cancer cells; this was especially true for *ERLIN2*, a lipogenic gene found in breast cancer cells [26, 27]. The most significantly increased saturated fatty acids were palmitic (C16:0) and stearic (C18:0) acids, and the most increased unsaturated fatty acid was arachidonic acid (C20:4) in breast cancer tissue, whose level increased with cancer progression [10]. We found that the levels of PS 18:0/20:4, PI 18:0/20:4, and PC 18:0/20:4 were markedly higher in highly metastatic MDA-MB-231 cells than in slightly metastatic MCF-7 cells, with no significant differences between MCF-7 cells and normal MCF-10A cells. Thus, those compounds can be suggested as potential biomarkers for metastatic potential of breast cancer.

Various predictive models and markers of cancer metastasis have been developed previously. Many recent studies have shown that levels of circulating tumor cells (CTCs) might be associated with the metastatic spread of various types of carcinomas [28, 29]. In addition, gene-expression profiling of breast cancer can be used to predict the risk of metastasis. A large amount of data has been generated for breast cancer, relating expression data to histological subtype [30, 31], site of tumor metastasis [32, 33], and metastatic potential [34, 35]. Circulating cell-free DNA, which carries tumor-specific alterations (circulating tumor DNA), was recently investigated in advanced cancer patients as an early-stage diagnostic biomarker [36, 37]. In present study, we demonstrated a PLSR model to predict metastasis of breast cancer based on metabolic and lipidomic profiles for the first time. Since whole data of metabolites and lipids are used for modeling, the PLSR model established with multivariate leads to better precisive and accurate prediction than univariate models.

Metabolomic analysis is complementary to genomic and proteomic approaches, and is being used to discover prognostic biomarkers in cancer. The present study simultaneously performed comprehensive profiling of alcohols, amino acids, fatty acids, organic acids, purines, sugars, and intact lipid species in breast cancer cells with different metastatic potentials. A predictive model was also developed. This resulted in adenine, guanine, xanthine, glucose-6-phosphate, and mannose-6-phosphate being identified as the novel potential biomarkers for the detection of the extent of breast cancer metastasis. In addition, this is the first report of the levels of PS 18:0/20:4, PI 18:0/20:4, and PC 18:0/20:4 being higher in highly metastatic cells (MDA-MB-231 cells) than in slightly metastatic cells (MCF-7 cells). Further study of a large cohort of breast cancer patients with different metastatic potentials should be conducted to verify these biomarkers.

The PLS regression model adopted in this study was able to successfully differentiate normal, slightly metastatic, and highly metastatic breast cancer cells with a high predictive accuracy. To establish an accessible and easily applicable data set for this predictive model, it is necessary to isolate primary human mammary cells from breast tissue taken from breast cancer patients. Breast tissues are generally dissociated by enzymatic digestion (most efficiently when using collagenase III), and then a differential centrifugation technique is applied to obtain the separated cells. The pellet consisting of epithelial cells can be utilized for metabolic profiling to predict the metastasis stage of breast cancer [38–40]. The metabolic and lipidomic approaches of this study provide an in-depth understanding of metabolism in breast cancer metastasis. It also provides valuable new information for assessing the metastatic potential of breast cancer in patients and for developing potential therapeutic agents.

## MATERIALS AND METHODS

### Chemicals and reagents

Butylated hydroxytoluene (BHT), myristic-d_27_ acid, methoxylamine hydrochloride, and pyridine were purchased from Sigma-Aldrich (St. Louis, MO). BSTFA [N,O-Bis(trimethylsilyl) trifluoroacetamide] containing 1% TMCS (trimethylchlorosilane) was purchased from Alfa Aesar (Ward Hill, MA). HPLC grade methanol, chloroform, and water were purchased from Fisher Scientific (Pittsburgh, PA). HPLC grade hexane was purchased from Honeywell Burdick & Jackson (Muskegon, MI).

### Cell culture

Human breast cancer cell lines MCF-7 and MDA-MB-231 (American Type Culture Collection, Manassas, VA) and the immortalized normal breast epithelial cell line, MCF-10A (American Type Culture Collection, Manassas, VA), were used for the experiments. MCF-7 and MDA-MB-231 cells originated from a human invasive adenocarcinoma. MCF-7 cells have slightly metastatic potential and belong to the luminal A group, and are known to express estrogen and progesterone receptors. MDA-MB-231 cells are highly metastatic and belong to the triple-negative basal-like group. MCF-7 and MDA-MB-231 cells were cultured in RPMI medium supplemented with 10% (v/v) heat-inactivated fetal bovine serum and 1% penicillin-streptomycin (Hyclone labs, Logan, UT). MCF-10A cells were cultured in DMEM/F12 medium supplemented with 5% horse serum (ThermoFisher Scientific, Waltham, MA), 20 ng/mL epidermal growth factor (Peprotech, Rocky Hill, NJ), 0.5 mg/mL hydrocortisone (Sigma-Aldrich, St. Louis, MO), 100 ng/mL cholera toxin (Sigma-Aldrich, St. Louis, MO), 10 μg/mL insulin (Sigma-Aldrich, St. Louis, MO), and 1% penicillin-streptomycin at 37°C in a humidified incubator with 5% CO_2_. The cells were sub-cultured with a seeding density of 1 × 10^6^ cells/mL and were grown to approximately 90% confluence for metabolic and lipidomic profiling experiments.

### Harvesting cell samples

Cells were detached using a 1× trypsin solution at 37°C in a humidified atmosphere of 5% CO_2_. After incubation, MCF-10A cells were resuspended in resuspension media and centrifuged at 150 × *g* for 3 min; MCF-7 and MDA-MB-231 cell lines were resuspended in growth media and centrifuged at 185 × *g* for 3 min. The media was aspirated, and the cell pellet was washed twice with 1.5 mL ice-cold phosphate-buffered saline (PBS) to remove extracellular metabolites. After the final wash step, cells were suspended in PBS and transferred into 2 mL Eppendorf tubes. Cell suspensions were immediately frozen in liquid nitrogen and stored at −80°C until analysis.

### Sample preparation and metabolite extraction

The cell suspensions were lysed by two freeze-thaw cycles, followed by sonication on ice. Briefly, cells were thawed in a 4°C water bath, vortexed vigorously, and sonicated for 20 min. They were then transferred to liquid nitrogen for 60 min, thawed in a 37°C water bath for 30 min, and briefly vortexed. This freeze-thaw cycle was repeated once for complete cell disruption, and was followed by an additional sonication for 10 min. Protein concentrations were determined using the Bio-Rad protein assay kit (Thermo Scientific, Rockford, IL) with bovine serum albumin (BSA) standards for normalization. The samples were centrifuged at 18,500 × *g* for 10 min at 4°C, dried in a freeze dryer (IlShinBioBase, Dongduchun city, Kyunggi-do, Korea), and stored at −70°C until analysis. Each sample was prepared and analyzed in quintuplicate.

The samples were extracted using a modified Folch procedure [41]. In brief, ice-cold chloroform (1 mL) and methanol (0.5 mL), with 0.1% BHT, were added to the dried cells and vortexed for 20 sec. The mixture was then sonicated in a 4°C water bath for 30 min and incubated on ice for 60 min with shaking. Phase separation was induced by adding 380 μL of water with 0.1% BHT, followed by incubation on ice for 10 min with shaking. The mixture was then centrifuged at 18,500 × *g* for 10 min at 4°C. The mixture was split into two aliquots, the upper (methanol) phase for GC-MS analysis, and the lower (chloroform) phase for lipid analysis using nano electrospray ionization mass spectrometry (nanoESI-MS). The extracts were passed through 0.2 μm PTFE syringe filter (Whatman, Maidstone, UK) and then evaporated with nitrogen gas. The dried chloroform fraction was resuspended in 100 μL of methanol-chloroform (9:1, v/v) containing 7.5 mM ammonium acetate buffer solution for analysis, and the dried methanol fraction was used for derivatization procedures. All extracts were stored at −80°C before analysis.

### GC-MS analysis

To perform derivatization of the extracted sample, 30 μL of 20,000 μg/mL methoxylamine hydrochloride in pyridine, 50 μL of BSTFA containing 1% TMCS, and 10 μL of myristic-d_27_ acid (500 μg/mL as an internal standard) were added to dried samples. The samples were then incubated for 60 min at 65°C. Finally, each extracted sample was used for GC-MS analysis.

GC-MS analysis was performed using a 7890A GC (Agilent Technologies, CA) model equipped with an autosampler (7683 B series, Agilent Technologies), and a 5975C mass selective detector (Agilent Technologies, CA). An injector volume of 1.0 μL was injected into a split/splitless inlet at 250°C. Helium was used as the carrier gas with a constant flow rate of 1.0 mL/min. A fused silica capillary column of 5% phenylmethylpolysiloxane phase (DB-5, Agilent Technologies) with dimensions 30 m × 0.25 mm i.d. × 0.25 μm film thickness was used for analysis. The auxiliary, MS source, and MS quad temperatures were set to 280, 230, and 150°C, respectively. The mass range was 50–700 Da, and data were obtained in full scan mode. Electron impact ionization mode, with ionization energy of 70 eV and a split ratio of 1:10, were used for GC-MS detection. The initial oven temperature was set to 70°C and was programmed to increase to 190°C (at 5°C/min) and then 240°C (at 6°C/min), and finally 280°C (at 5°C/min). The metabolites were identified by comparison of mass spectra with those of NIST-Wiley Mass Spectra Library, Human Metabolome Database (HMDB; http://www.hmdb.ca/), and Golm metabolome Database (GMD; gmd.mpimp-golm.mpg.de/).

### Lipid analysis

Lipid extracts were analyzed in positive and negative ion modes via nanoESI-MS using a linear ion-trap mass spectrometer (LTQ-XL, ThermoFisher Scientific, San Jose, CA) equipped with an automated nanoinfusion/nanospray source (TriVersa NanoMateSystem, Advion Biosciences, Ithaca, NY). The spray parameters were set with a gas pressure of 0.2 psi and ionization voltage applied at 1.2 kV for positive and negative ion modes. The ion source was controlled using Chipsoft 8.3.1 software (Advion Biosciences). Aliquots (30 μL) were loaded into a 96-well plate, which was placed on a Nanomate cooling plate set to 4°C and 10 μL of each sample was infused into a mass spectrometer via an Advion ESI chip with 5.5 μm (inner diameter) emitter nozzles.

Full-scan spectra were collected in mass-to-charge ratio (m/z) ranges of 400–1200 and 500–1300, for positive and negative ion modes, respectively. The mass spectra of each sample were acquired in profile mode over a 2 min period. The capillary temperature was set to 200°C; the capillary and tube-lens voltages were set to 32 V and 95 V, respectively, in positive ion mode, and to −41 V and −93 V in negative ion mode. The target automatic-gain-control values for full MS and multistage MS were 30,000 and 1,000, respectively. MS/MS was applied to pooled samples for identification of lipid species. The normalized collision energy was set to 35%, with an isolated width of 1.5 m/z units and a charge state of 1. The dynamic exclusion parameters were a repeat duration of 60 sec, exclusion duration of 60 sec, and an exclusion list size of 50.

Lipid species were identified based on the MS/MS spectra of an authentic reference and an in-house MS/MS library. In addition, databases of Lipidmaps (http://www.lipidmaps.org/) and LipidBlast were used to match with spectra.

### Data acquisition and processing

The raw data files (*.raw) of lipids acquired from analyzed samples were converted into *.mzXML format using Proteo Wizard MSConvert [42]. Then, mass spectra were processed with Expressionist MSX software (version 2013.0.39, Genedata, Basel, Switzerland). The spectra scans were averaged and then spectrum smoothing, *m/z* alignment, and baseline subtraction were performed. Then, the data matrices were exported from Expressionist MSX as Excel files. For the relative quantification of metabolomes and lipidomes, relative levels of metabolites and lipids were calculated by dividing the peak intensity of the internal standard (PE 17:0/17:0) by the total protein content.

Fold changes, Student's *t*-tests (at a threshold of *p* <0.05), and pathway analysis were assessed using MetaboAnalyst (version 3.0; http://www.metaboanalyst.ca), a web-based software tool for metabolomics analysis. Significant differences were evaluated by a one-way analysis of variance (ANOVA) with a Tukey's *post-hoc* test using SPSS software (version 21, IBM, Somers, NY) and different letters indicate statistically significant differences between samples. The level of statistical significance was set at *p* <0.05.

For multivariate statistical analysis, partial least-squares discriminant analysis (PLS-DA) and partial least-squares projection to latent structures (PLS) regression (PLSR) were performed using SIMCA-P+ software (version 13.0, Umetrics, Umeå, Sweden) using mean-centered and unit variance-scaled data. The PLSR model was validated using the following steps: (i) cross-validation to assess the predictive power with *Q*^2^*Y* (predicted variation, or goodness of prediction) and *R*^2^*Y* (explained variation, or goodness of fit); (ii) response permutation testing to assess the statistical significance of the estimated predictive power with an *R*^2^*Y* intercept and *Q*^2^*Y* intercept on the validation plots; and (iii) external validation to test predictive performance by importing the lipid profiles of unknown samples [16].

## SUPPLEMENTARY MATERIAL FIGURES AND TABLES


