# Supplementary material for: Comparative metabolic and lipidomic profiling of human breast cancer cells with different metastatic potentials
Source: Oncotarget. 2016 Aug 24;7(41):67111–28. doi: 10.18632/oncotarget.11560 (PMC5341861; doi:10.18632/oncotarget.11560)
Supplement: Supplementary file 1 [file oncotarget-07-67111-s001.pdf]

# Comparative metabolic and lipidomic profiling of human breast cancer cells with different metastatic potentials

## SUPPLEMENTARY FIGURES AND TABLES

A

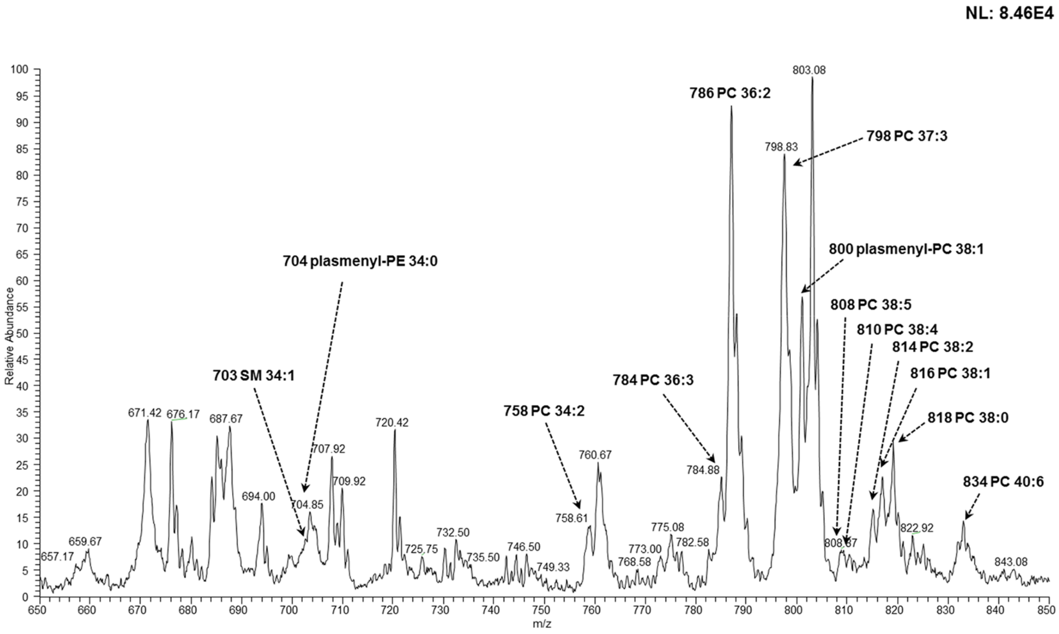


B

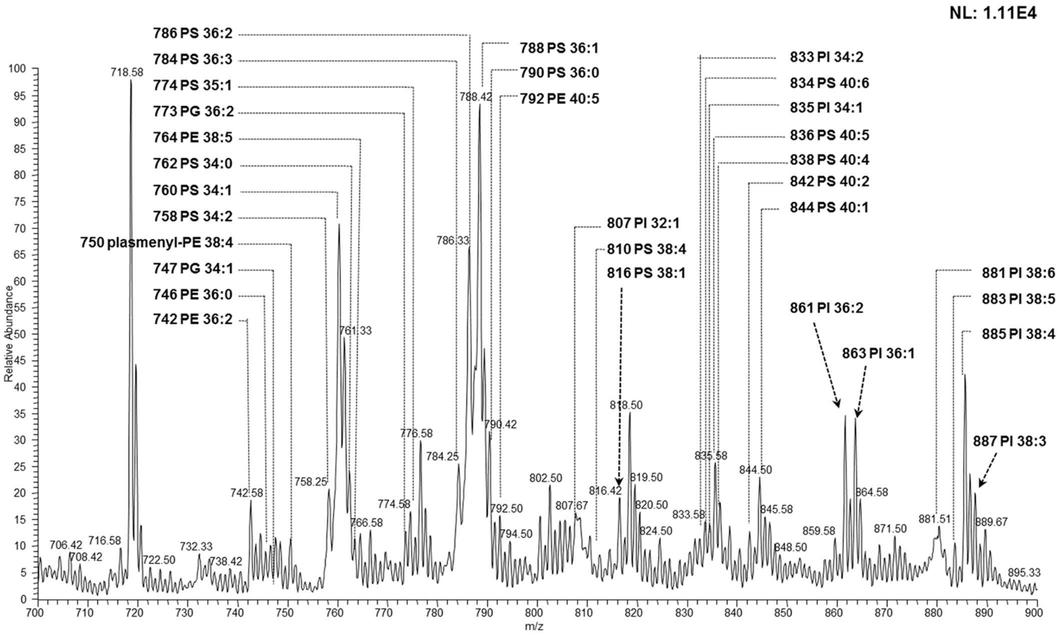


**Supplementary Figure S1: Representative spectra of lipid profiles from mammary epithelial and breast cancer cells, derived from nano-electrospray mass spectrometry in A. positive ion mode and B. negative ion mode.**

**Compound #1**

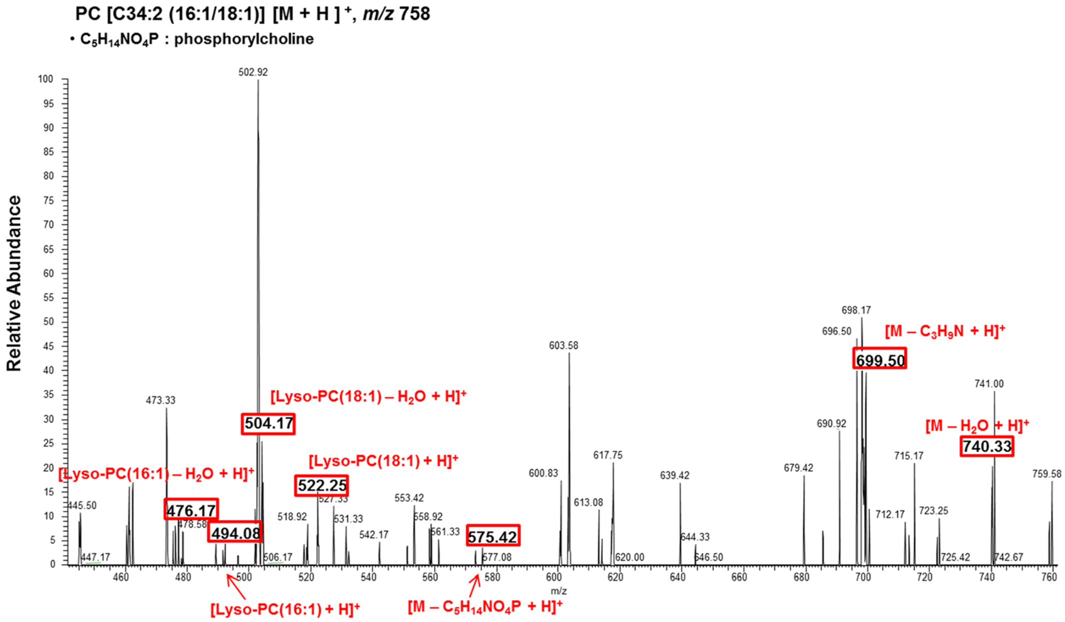


**Compound #2**

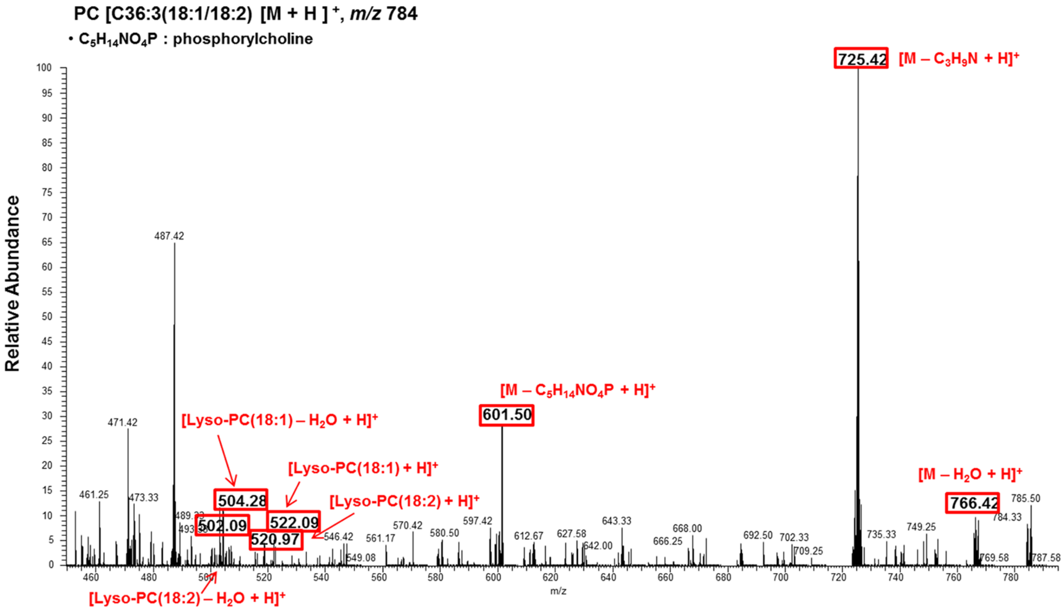


(*Continued*)

**Compound #3**

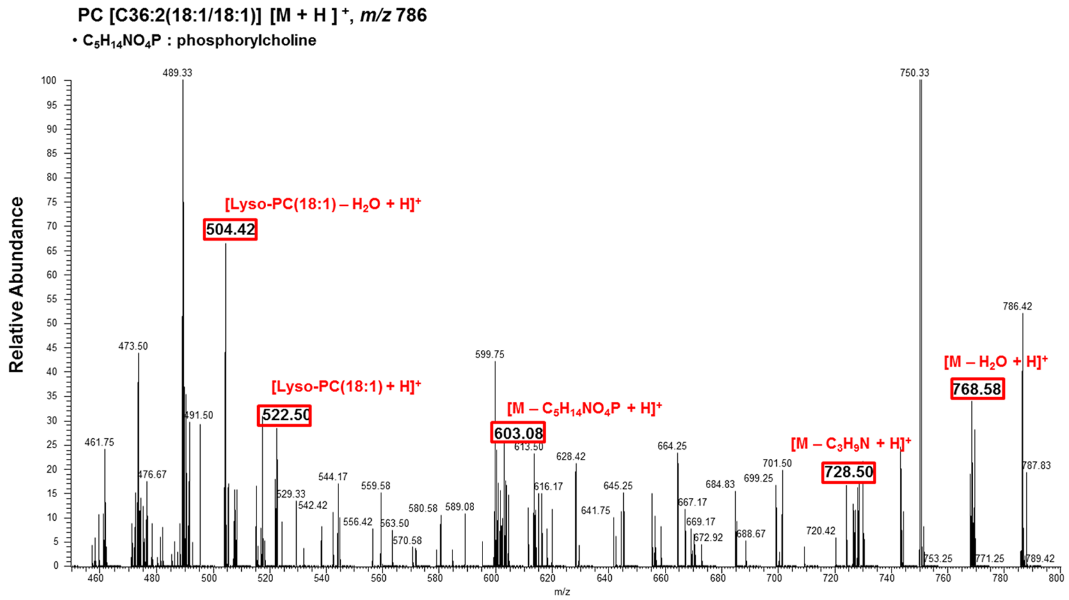


**Compound #4**

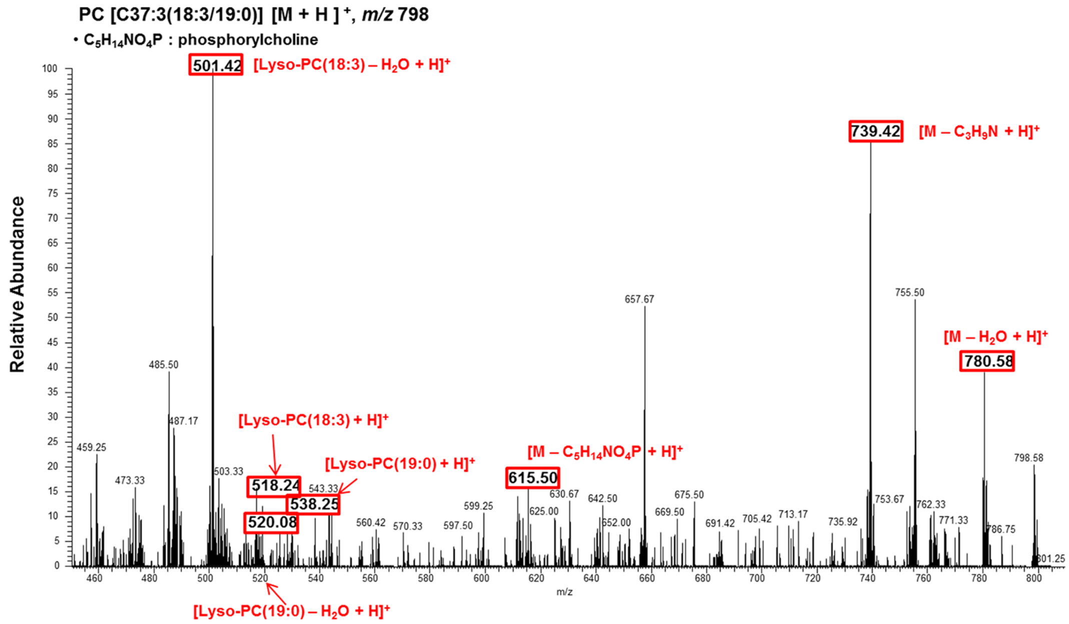


(*Continued*)

**Compound #5**

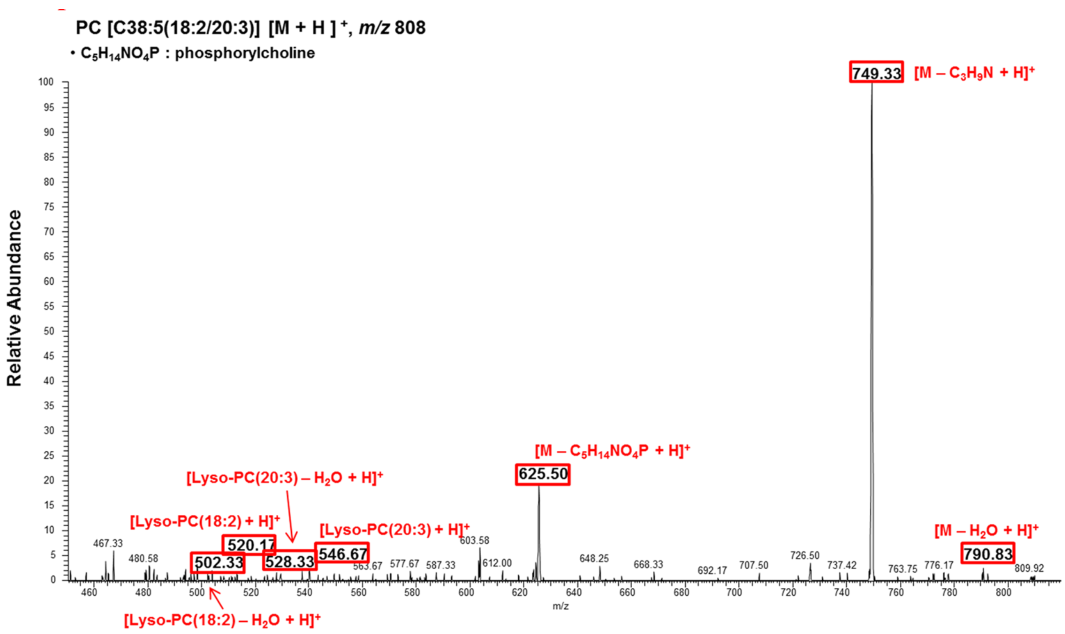


**Compound #6**

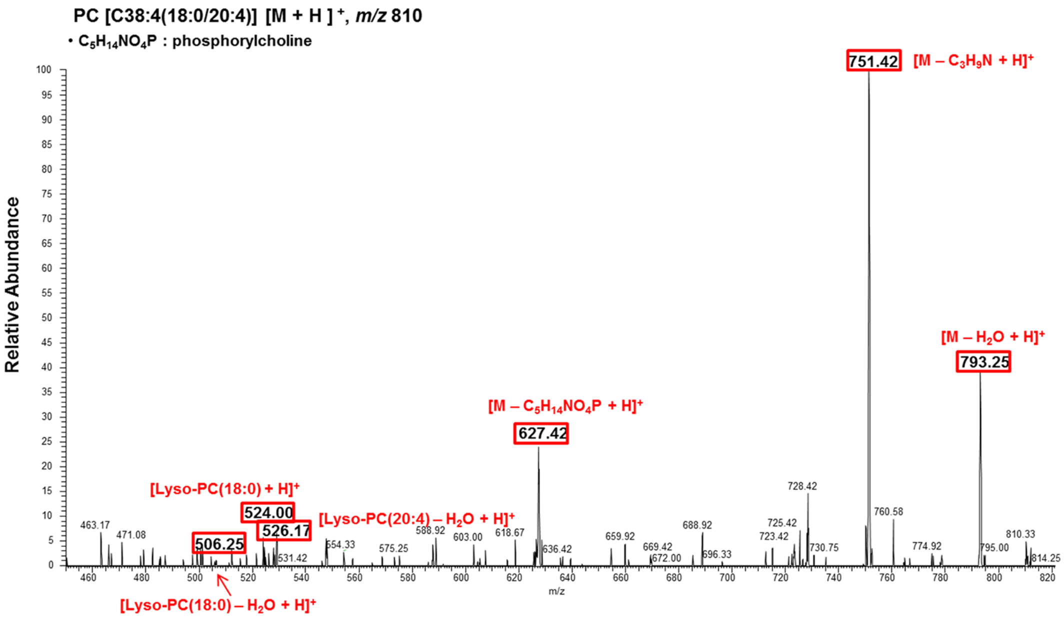


(*Continued*)

**Compound #7**

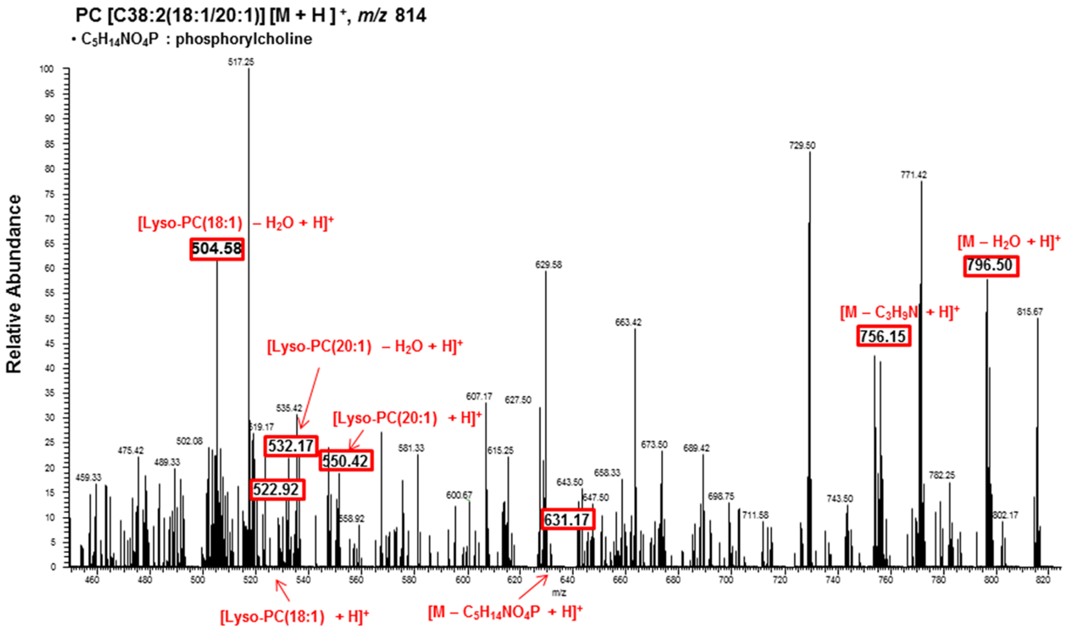


**Compound #8**

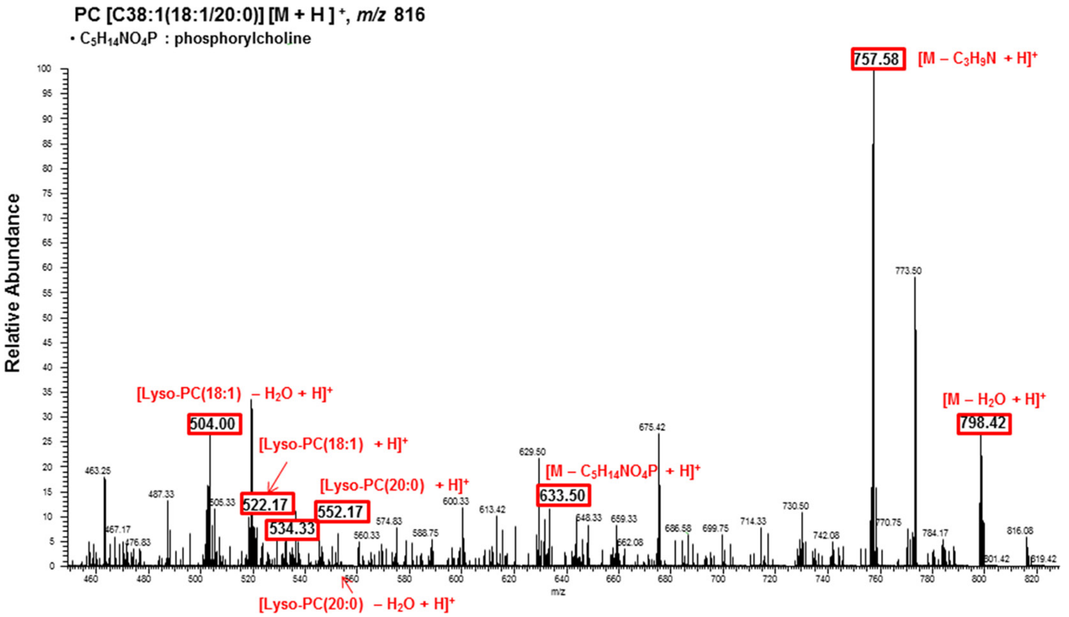


(*Continued*)

**Compound #9**

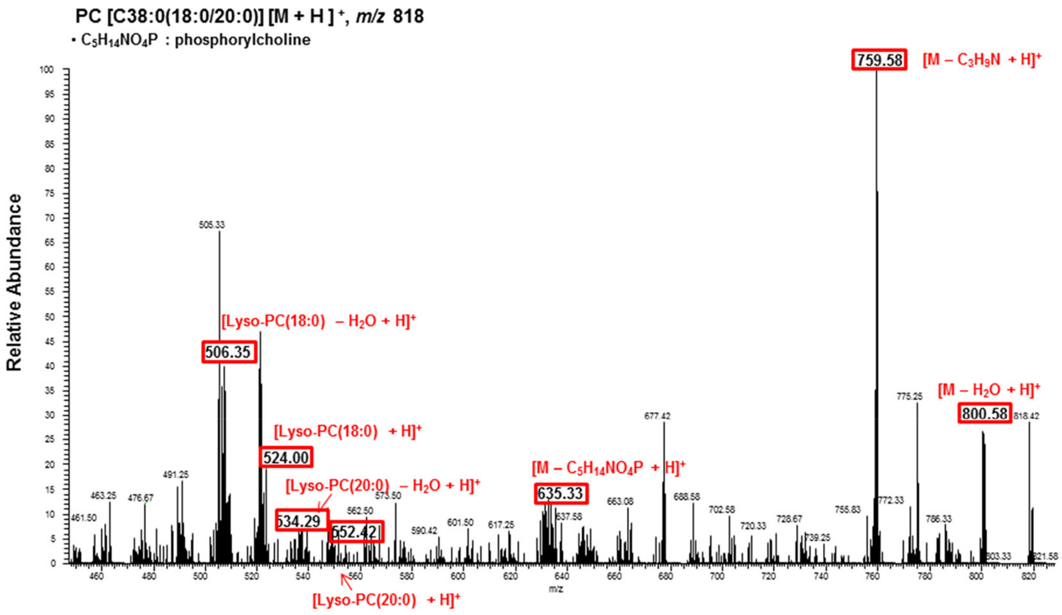


**Compound #10**

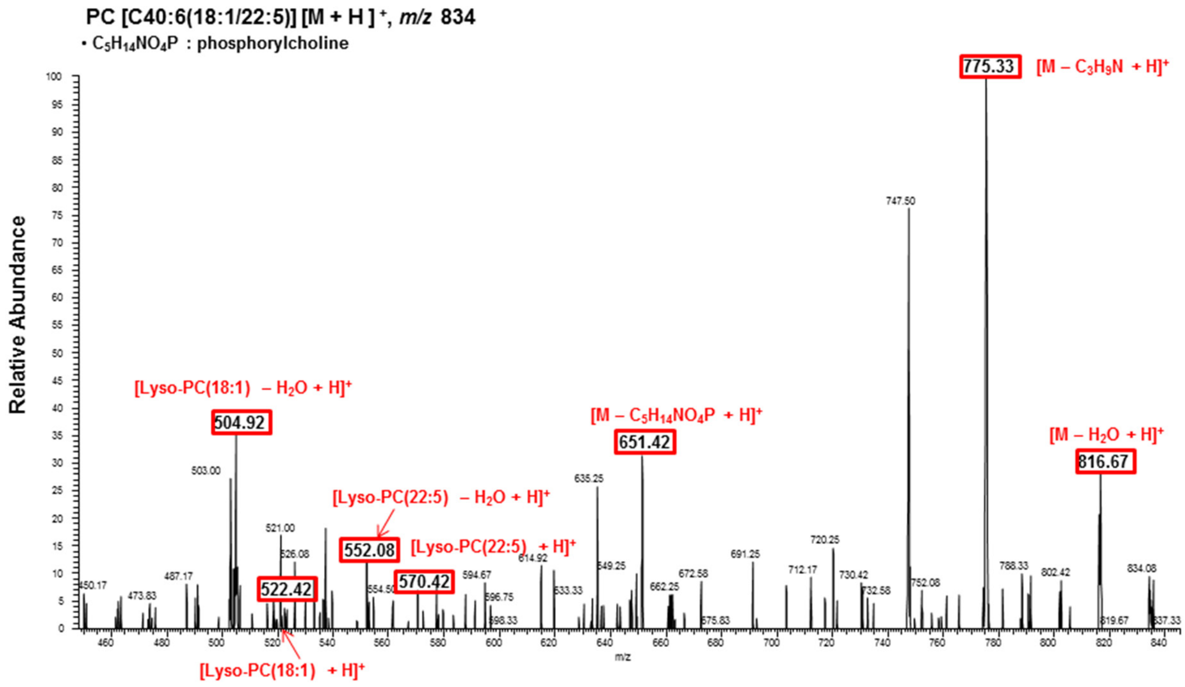


(*Continued*)

**Compound #11**

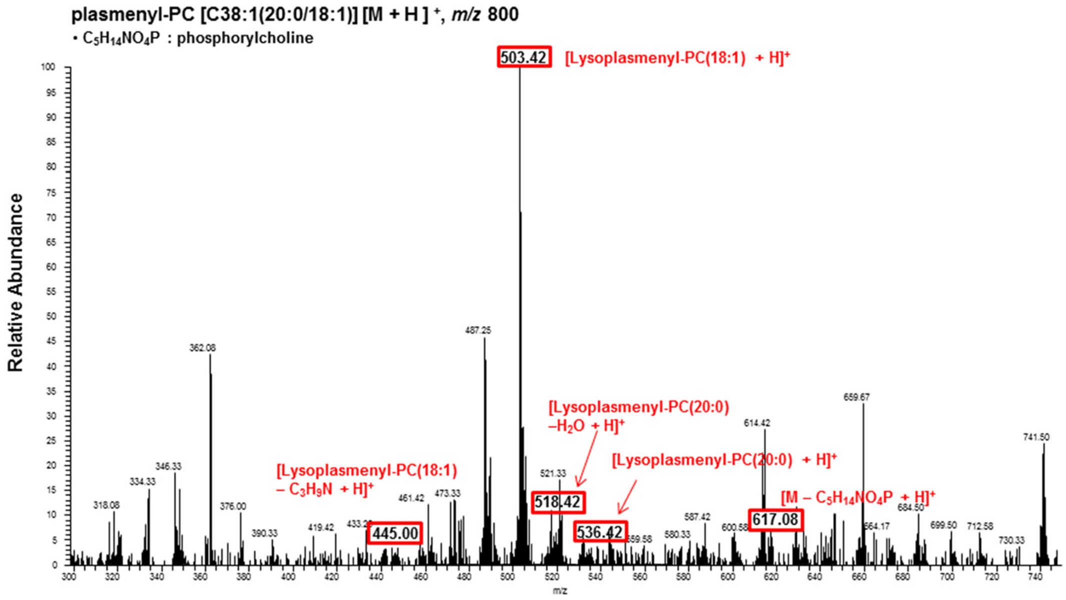


**Compound #12**

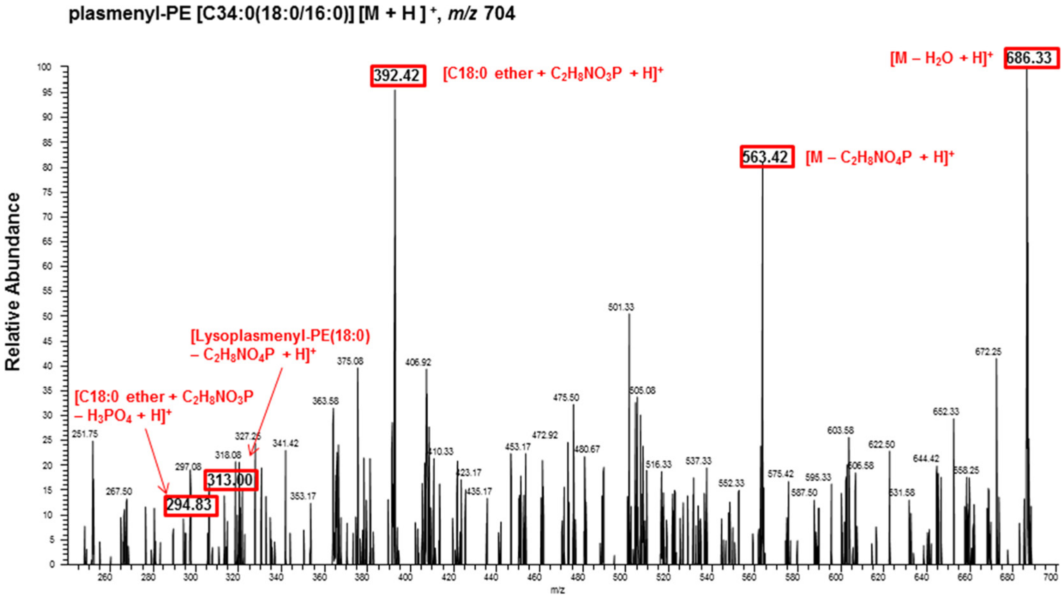


(*Continued*)

**Compound #13**

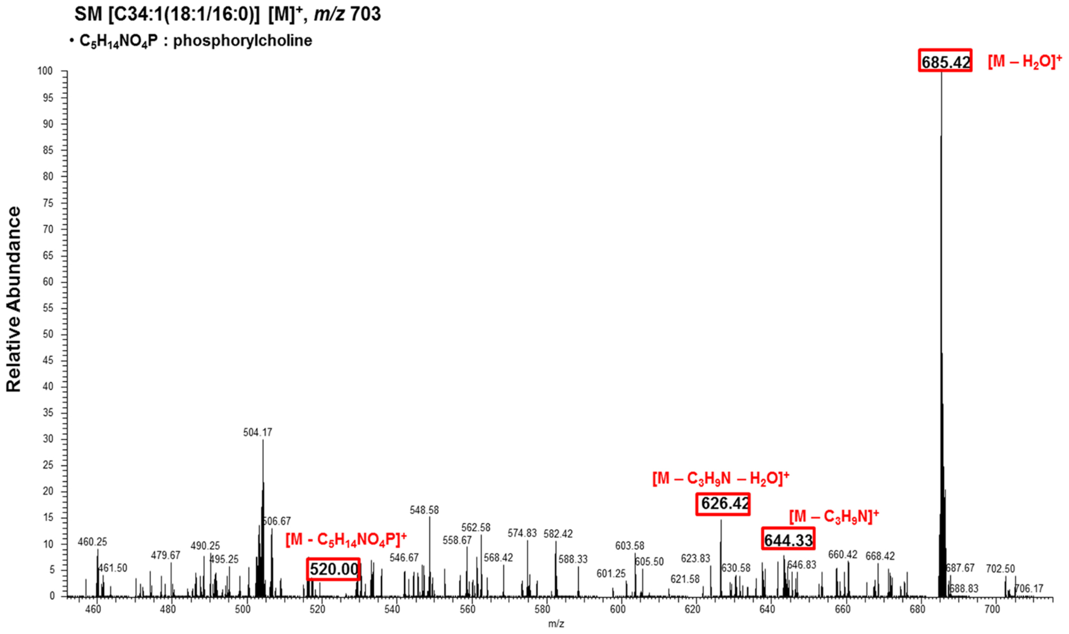


**Compound #14**

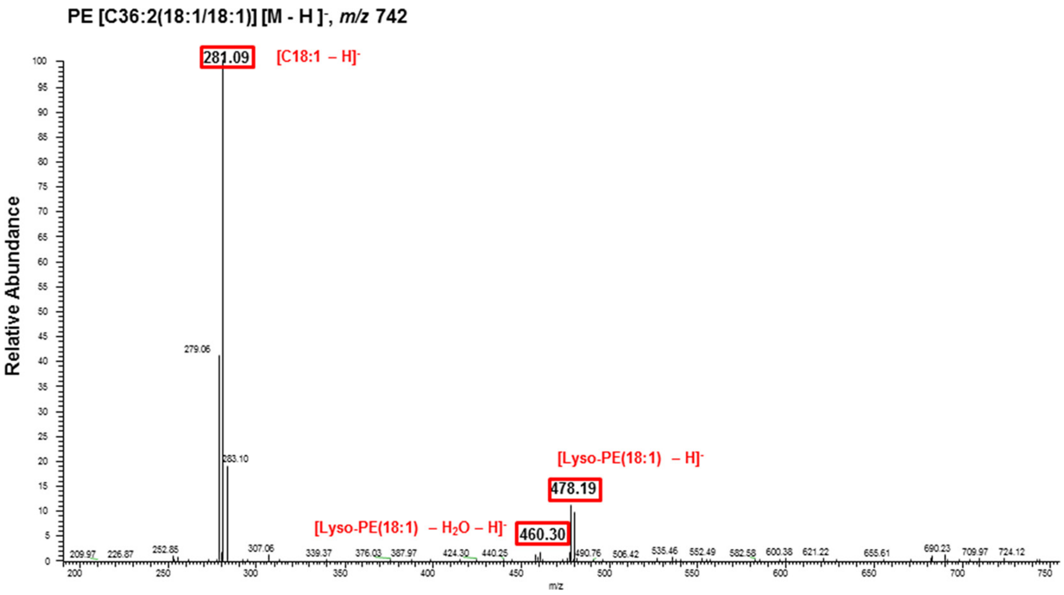


(*Continued*)

**Compound #15**

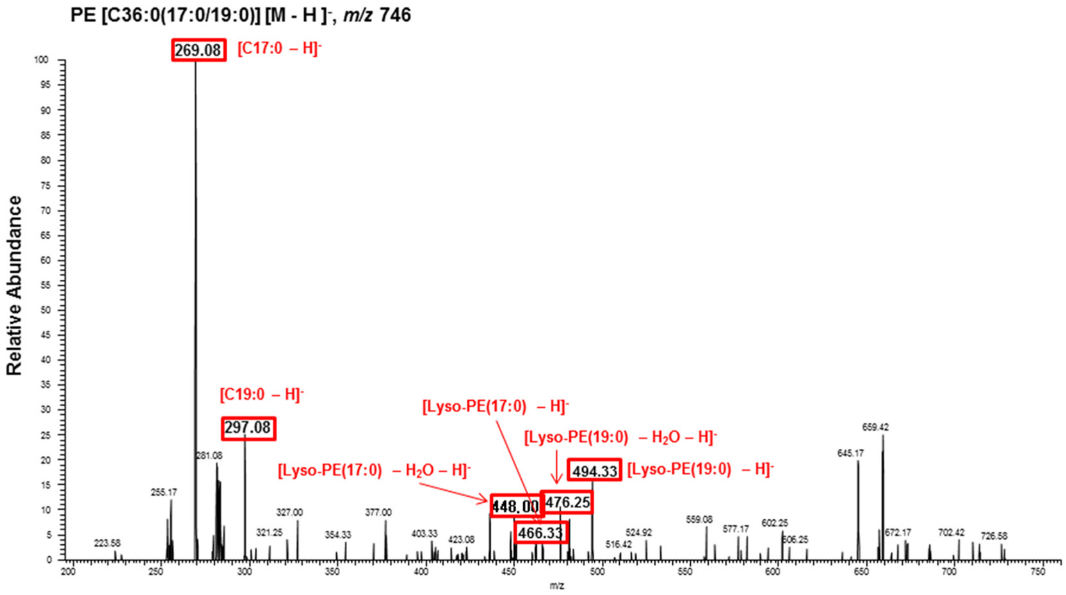


**Compound #16**

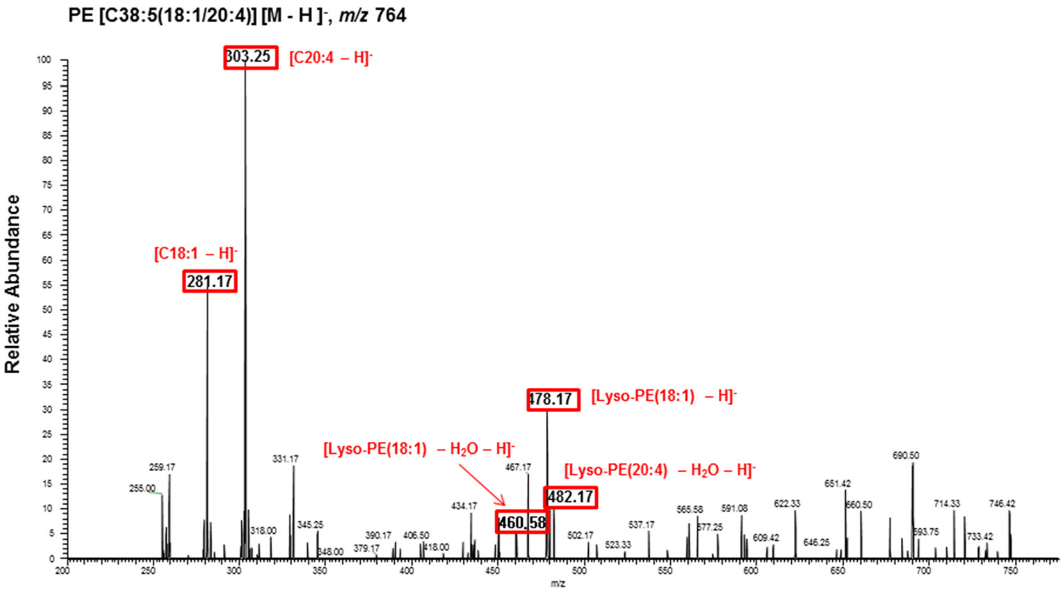


(*Continued*)

**Compound #17**

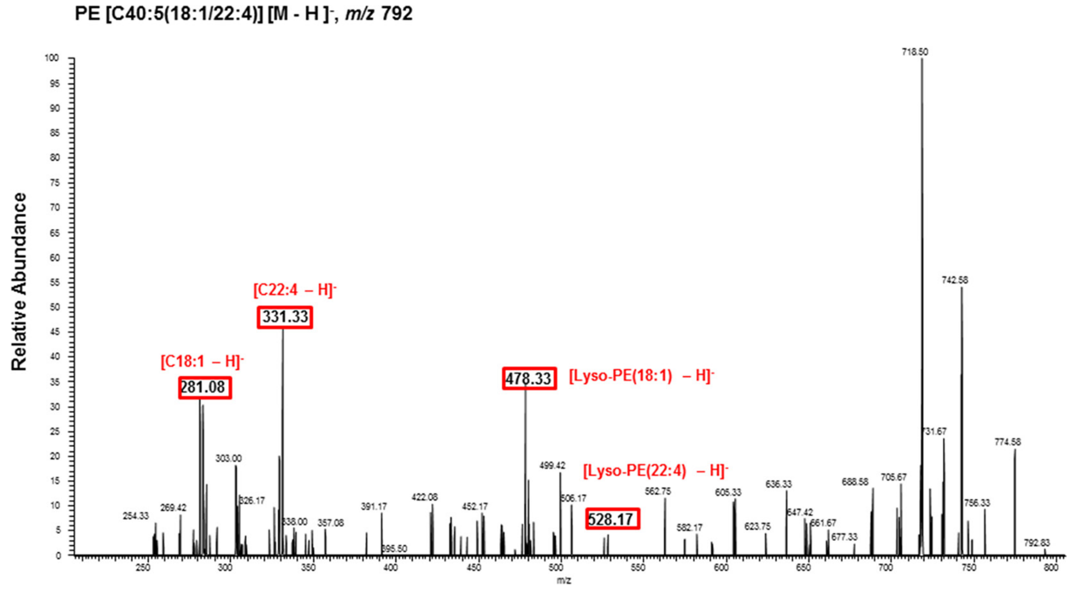


**Compound #18**

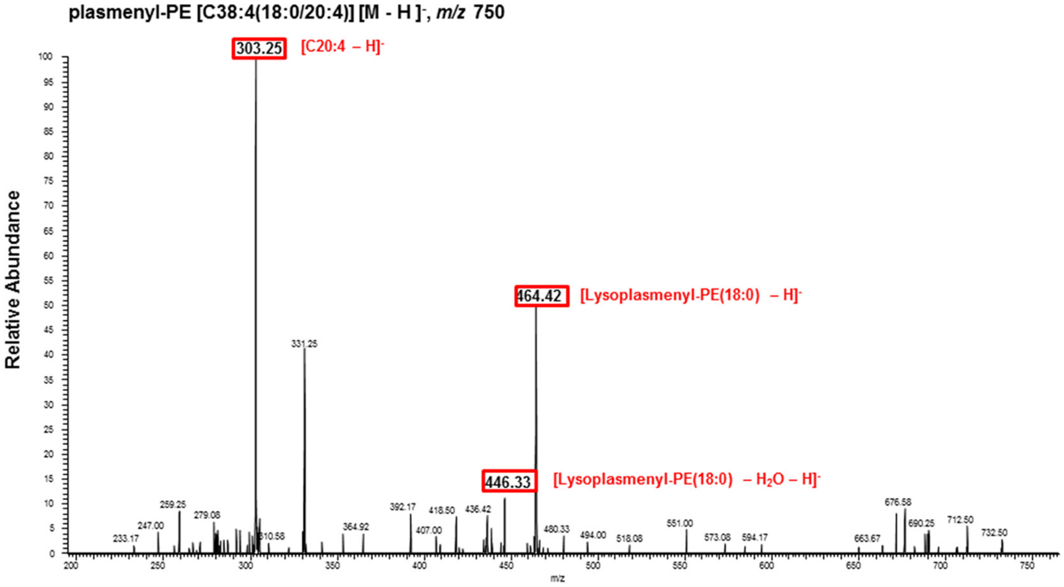


(*Continued*)

**Compound #19**

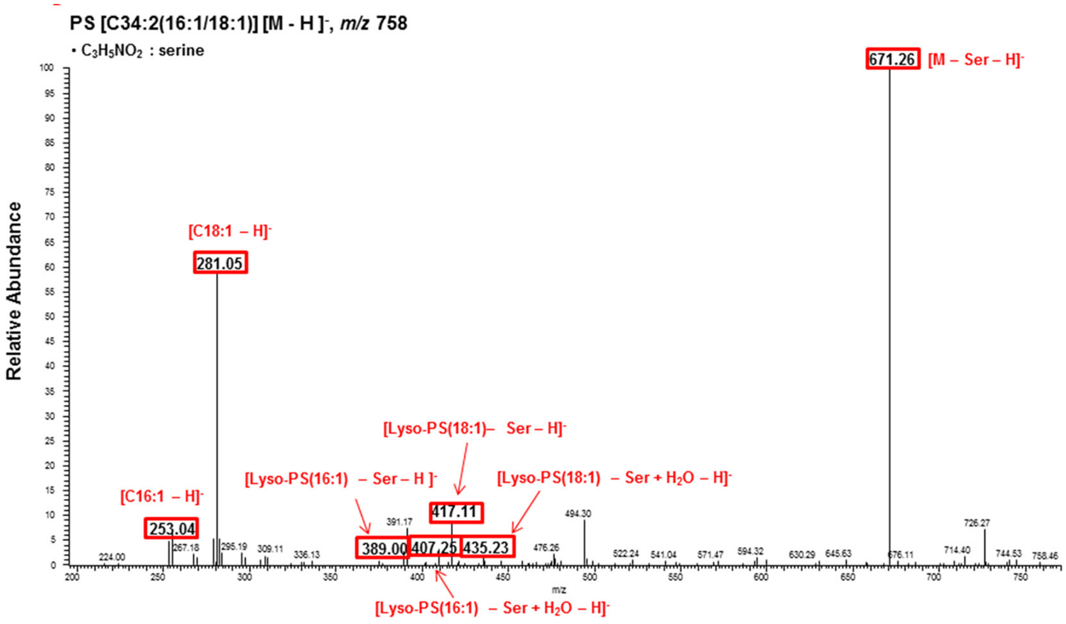


**Compound #20**

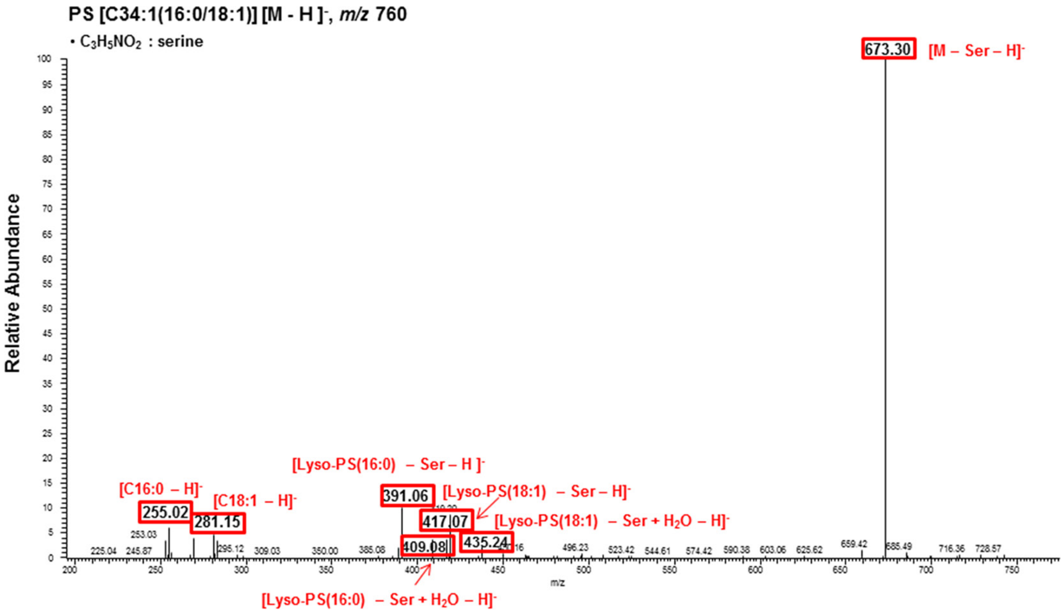


(*Continued*)

**Compound #21**

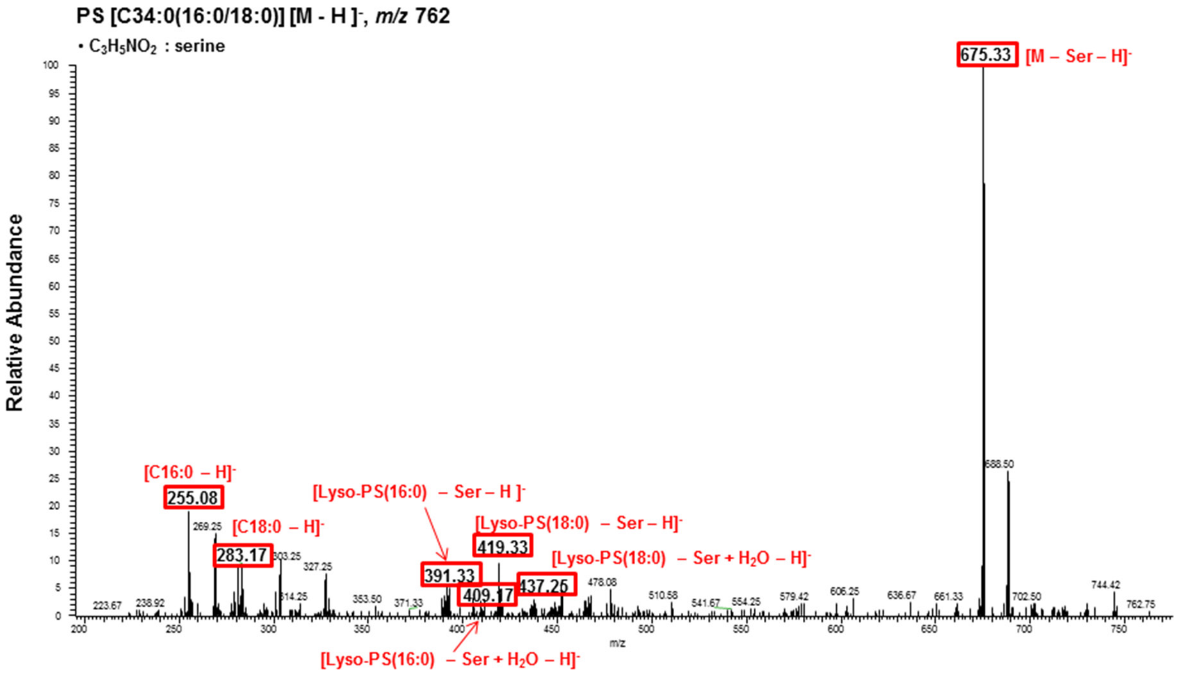


**Compound #22**

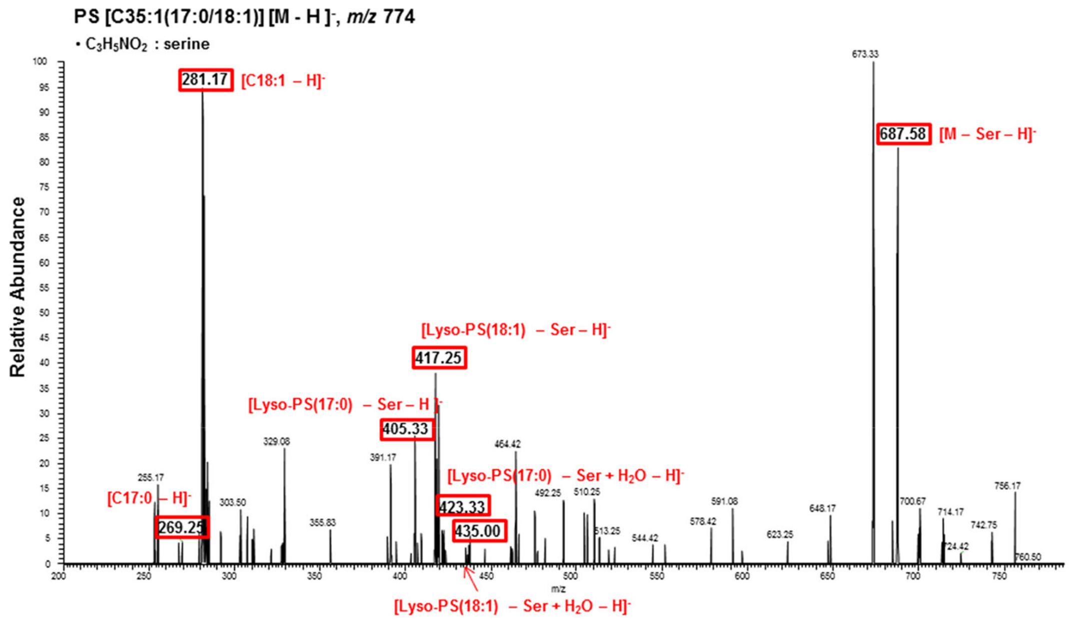


(*Continued*)

**Compound #23**

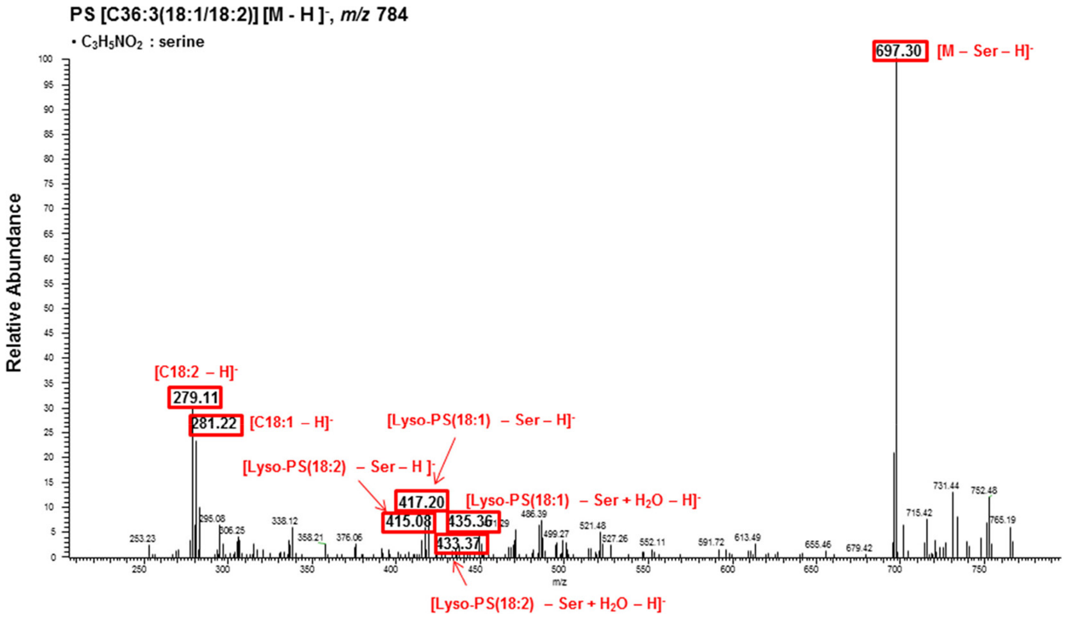


**Compound #24**

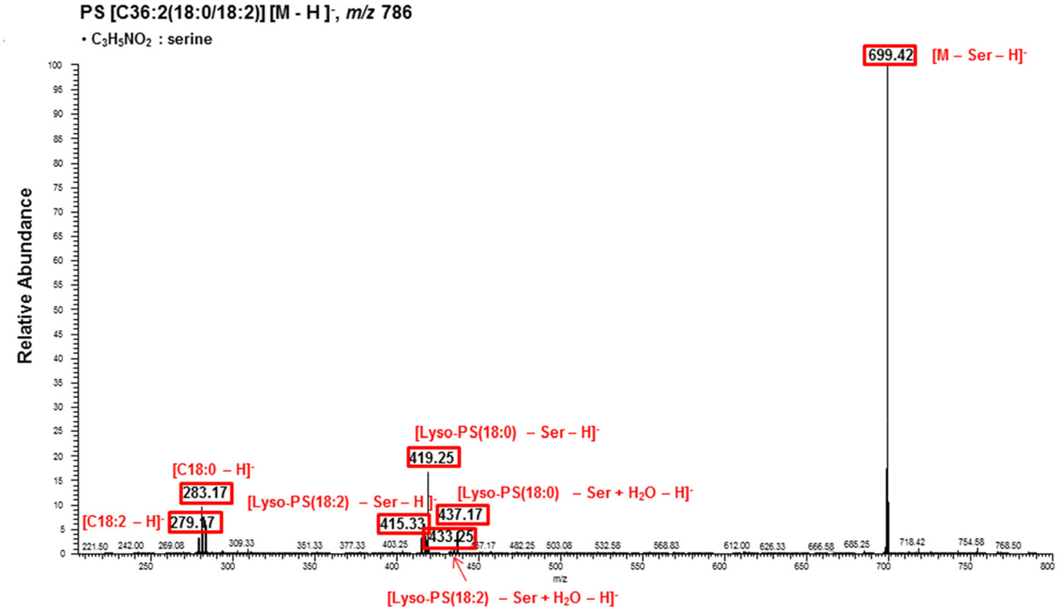


(*Continued*)

**Compound #25**

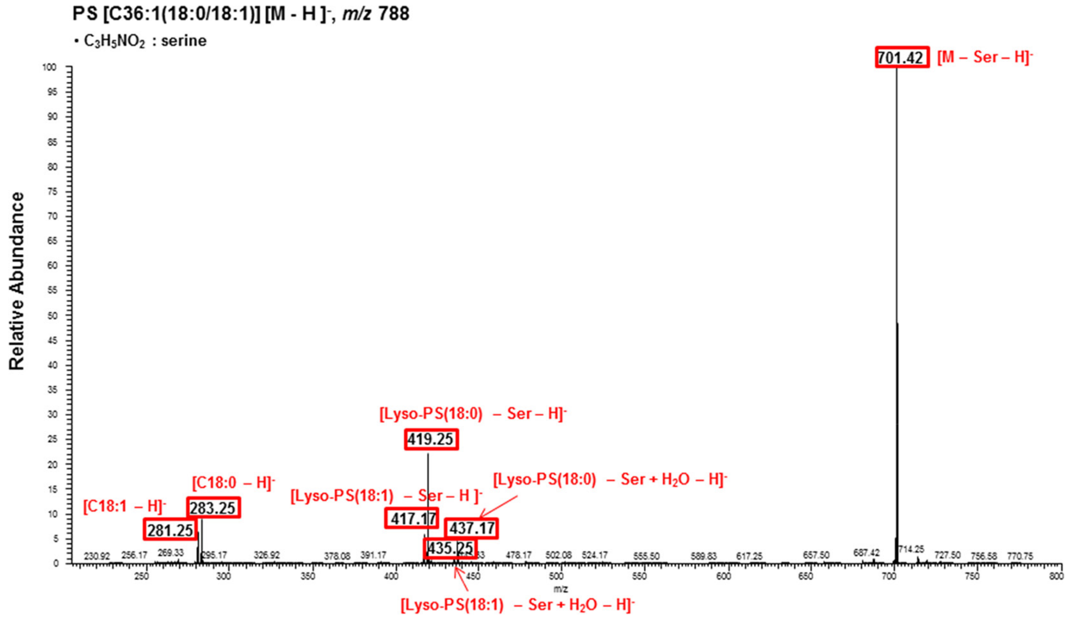


**Compound #26**

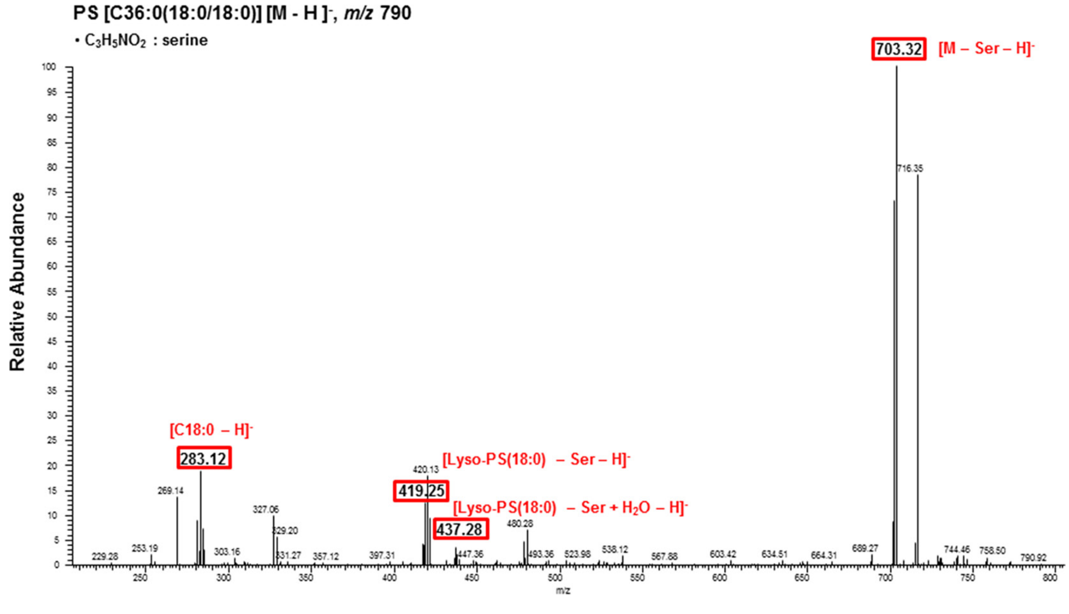


(*Continued*)

**Compound #27**

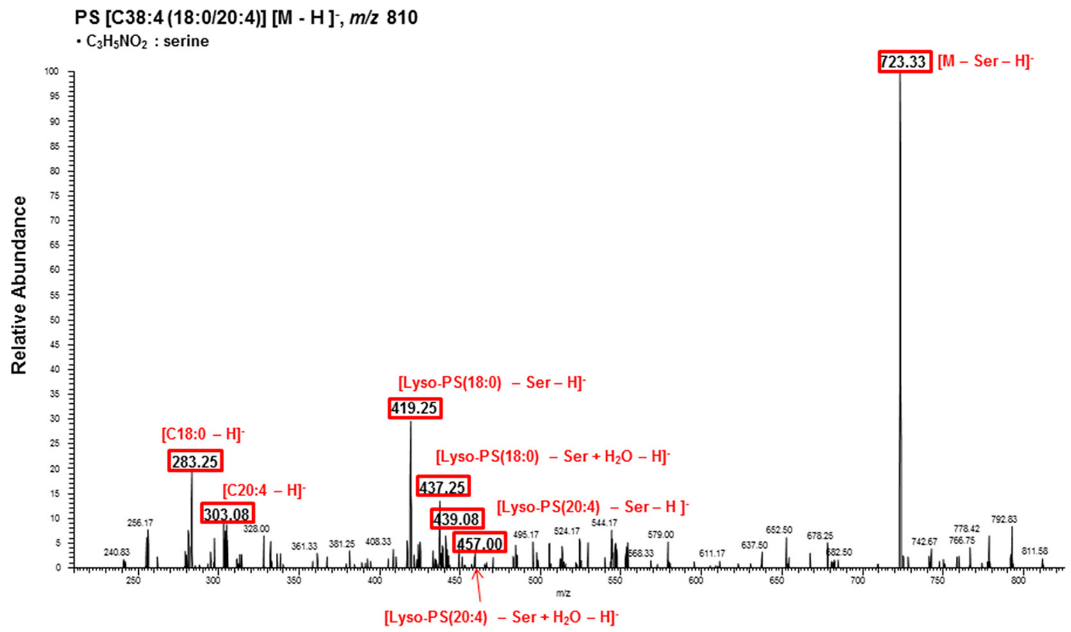


**Compound #28**

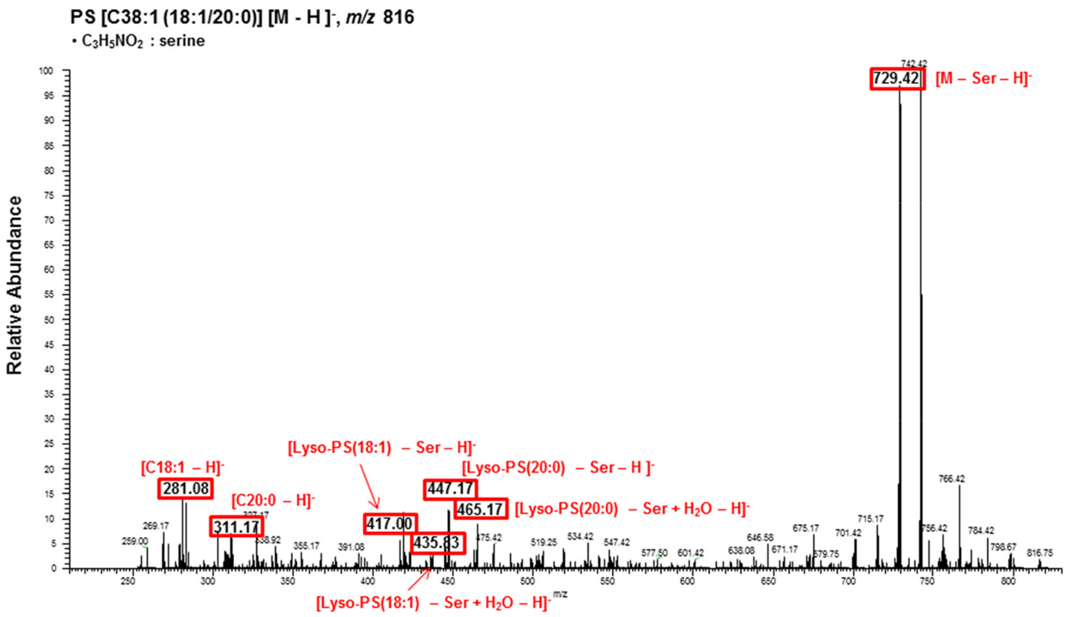


(*Continued*)

**Compound #29**

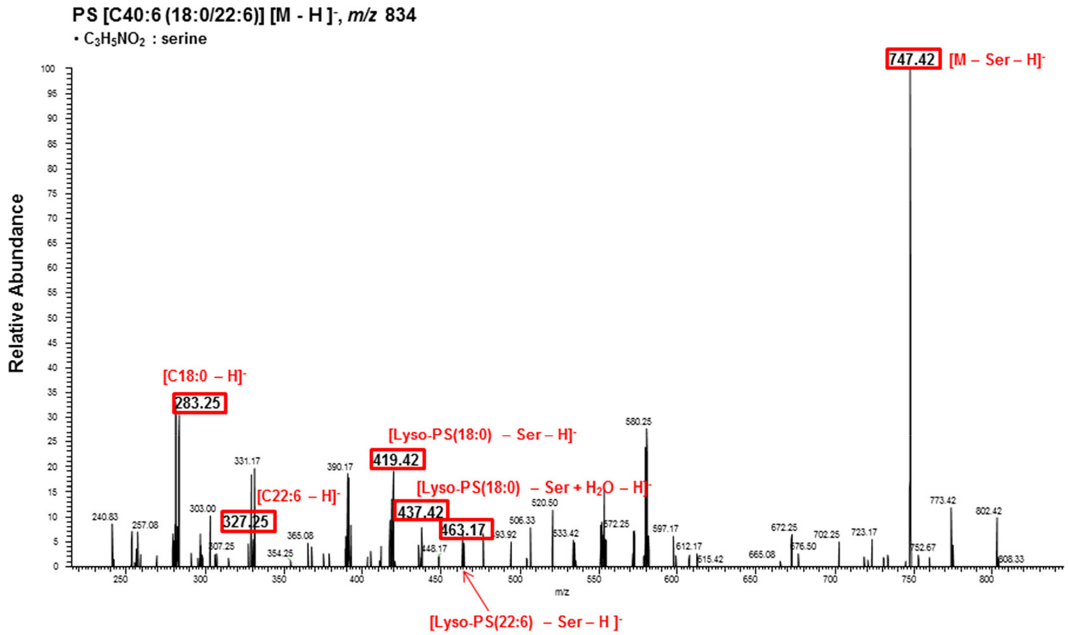


**Compound #30**

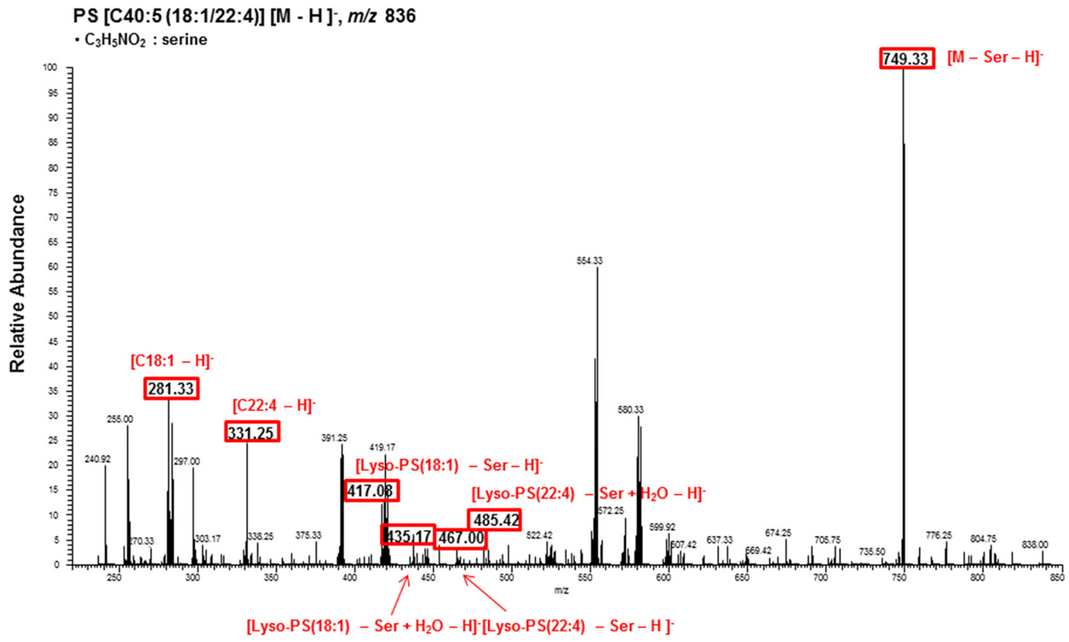


(*Continued*)

**Compound #31**

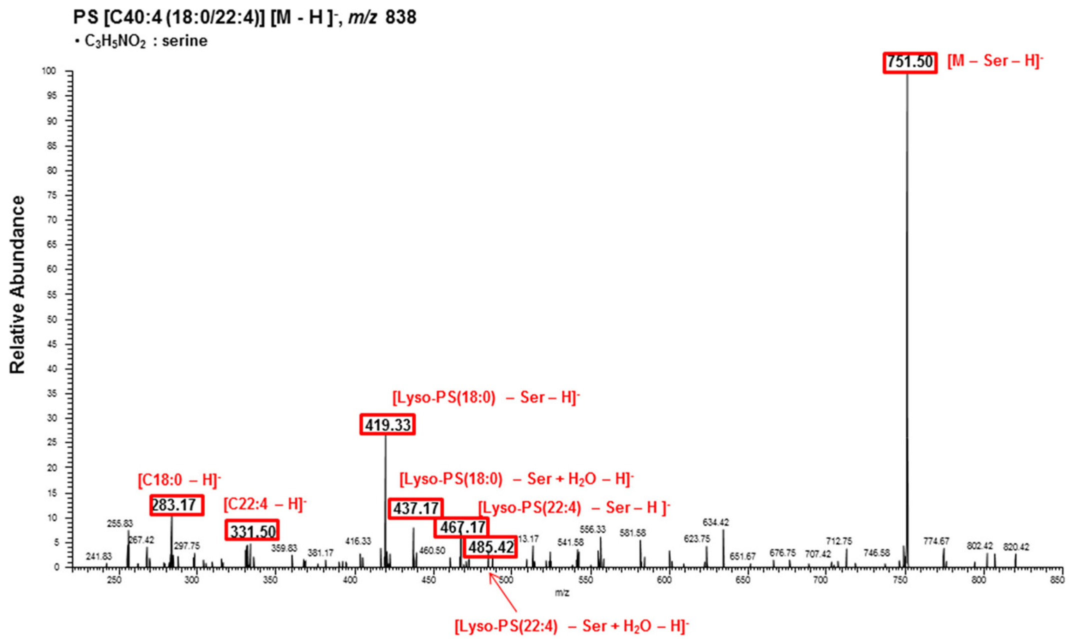


**Compound #32**

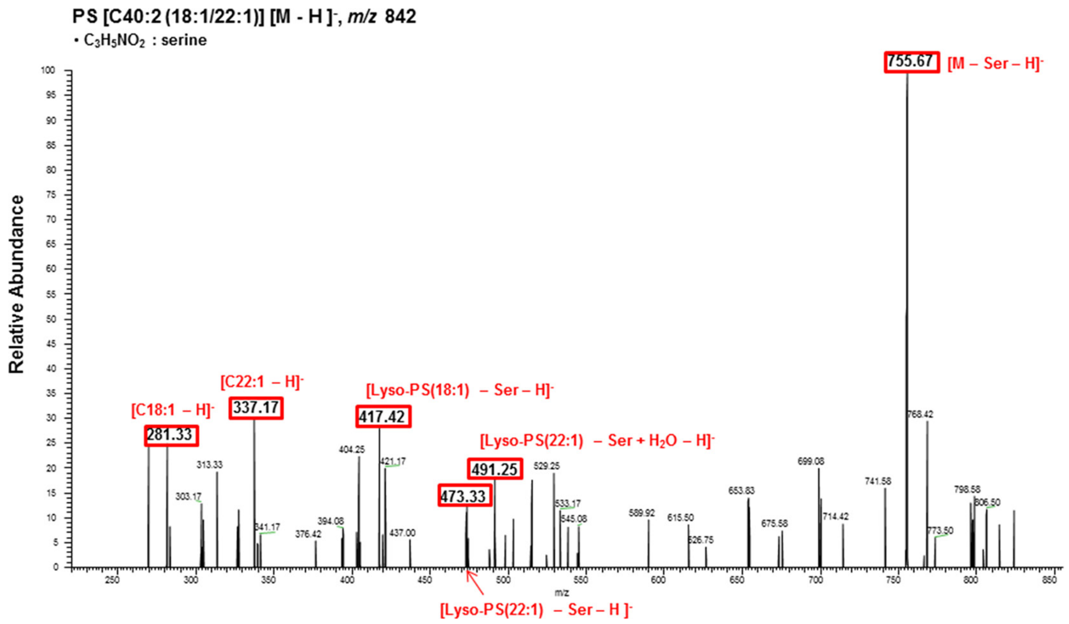


(*Continued*)

**Compound #33**

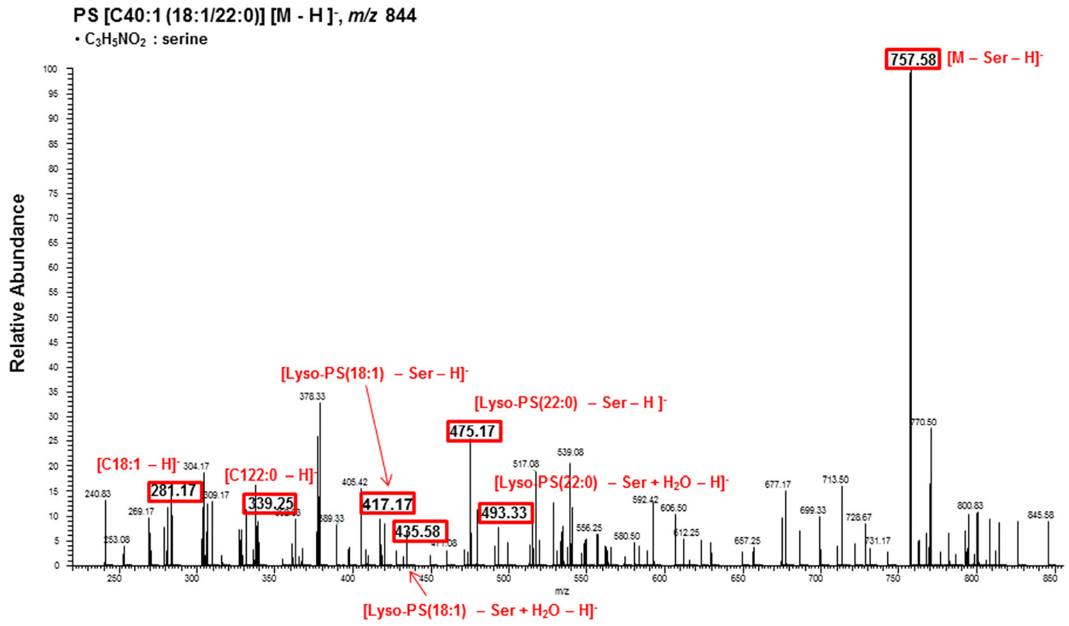


**Compound #34**

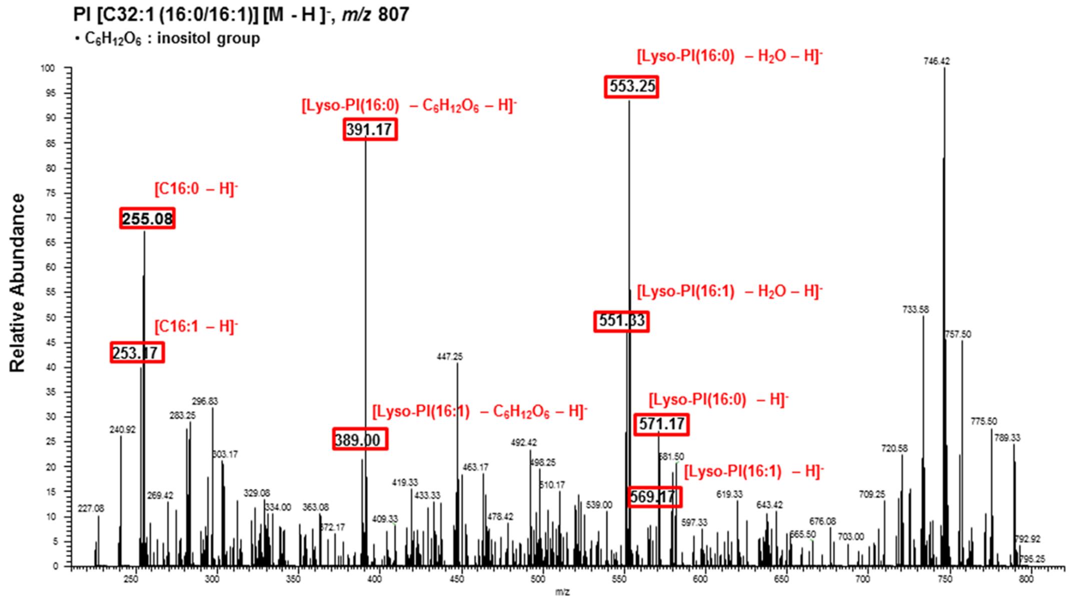


(*Continued*)

**Compound #35**

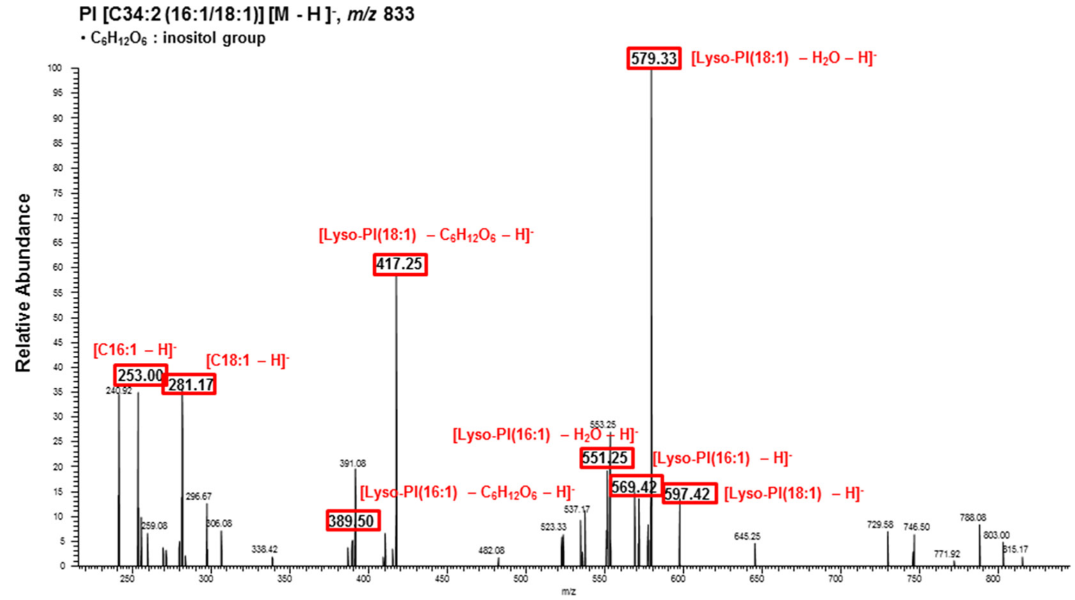


**Compound #36**

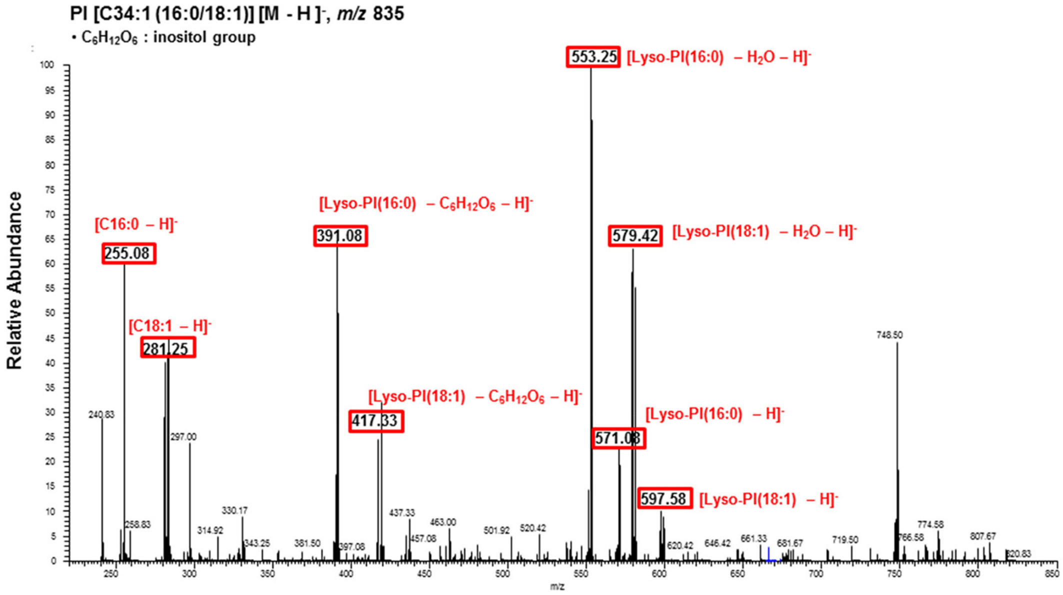


(*Continued*)

**Compound #37**

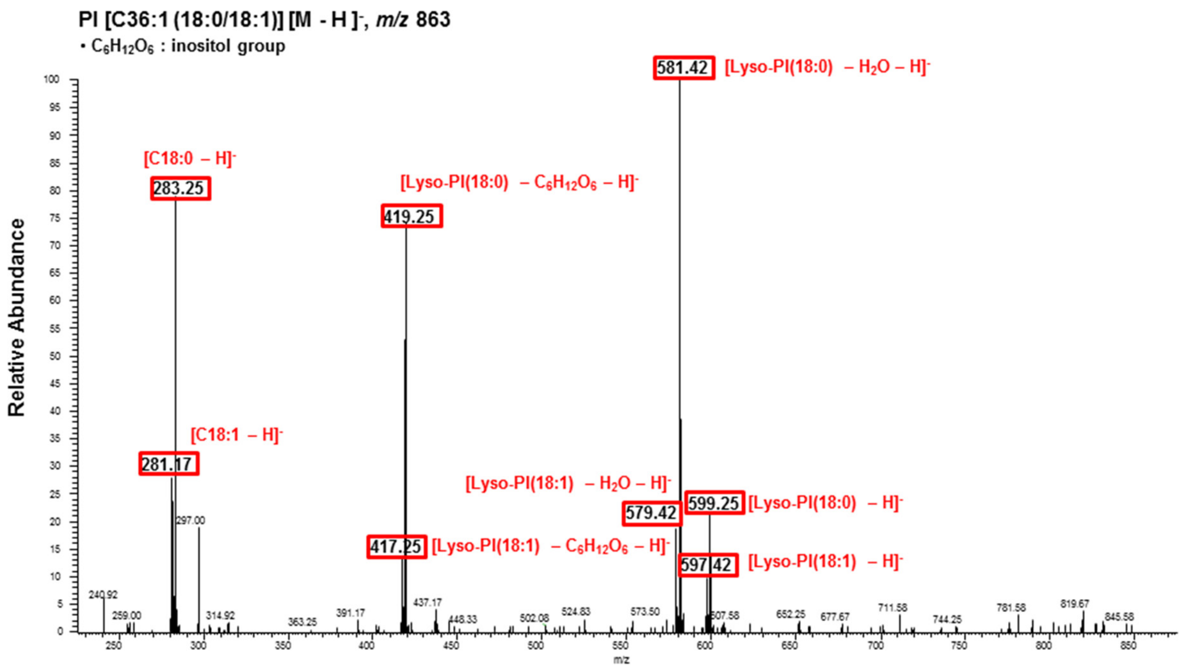


**Compound #38**

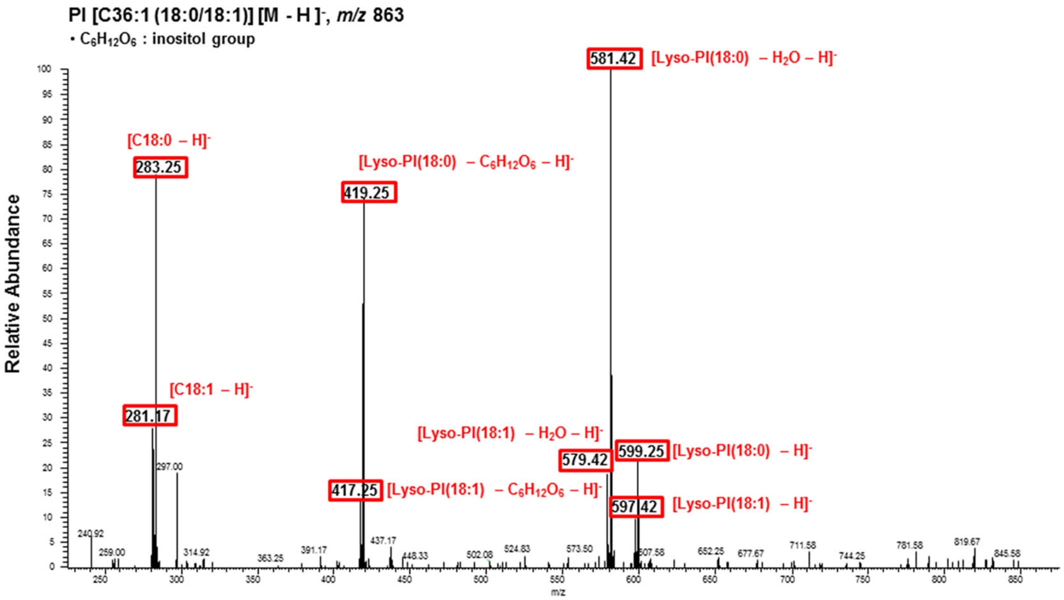


(*Continued*)

**Compound #39**

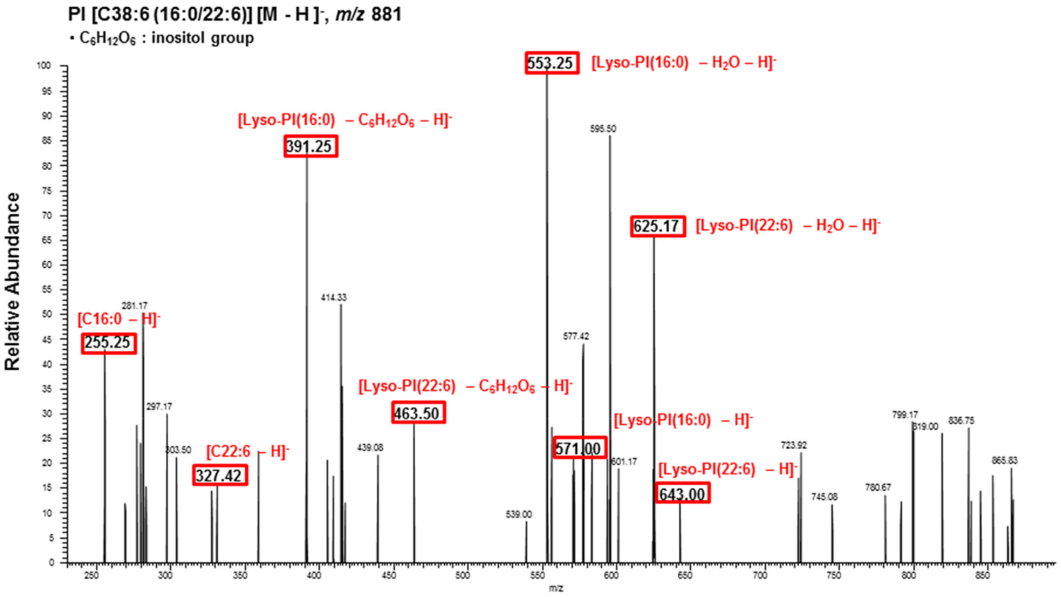


**Compound #40**

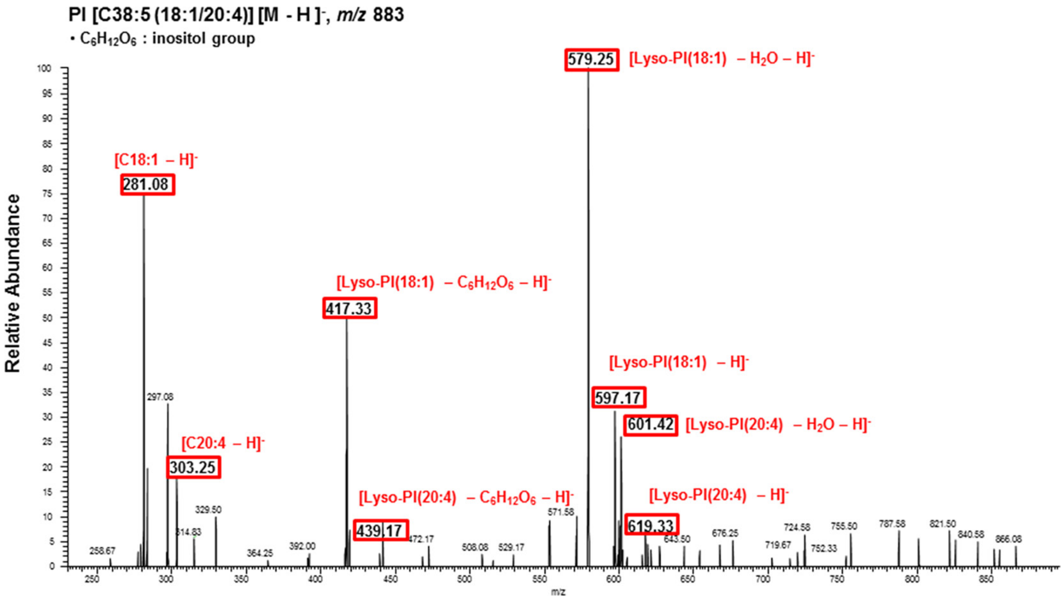


(*Continued*)

**Compound #41**

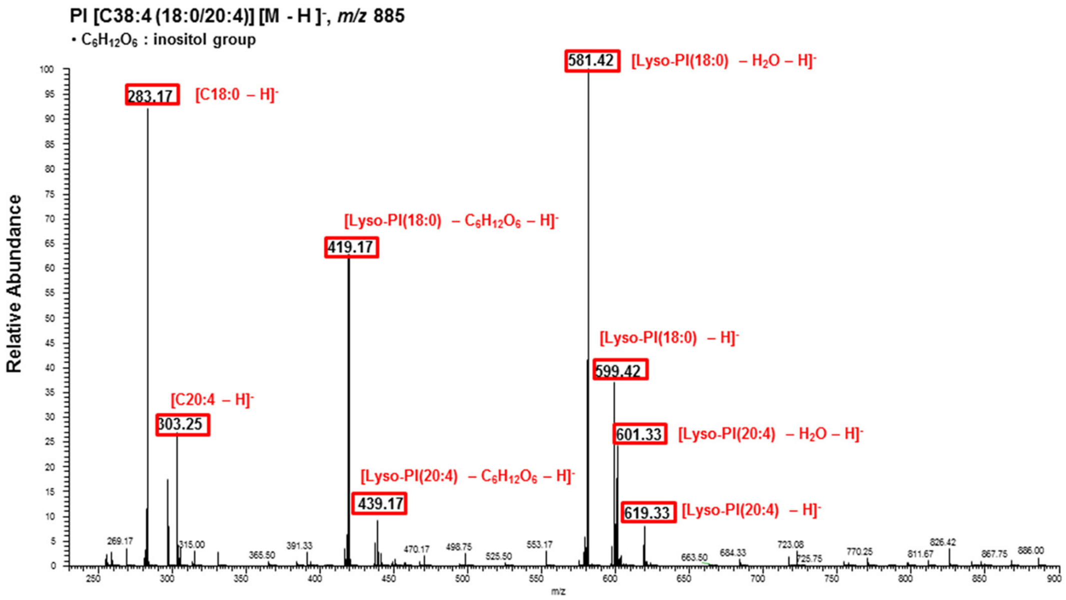


**Compound #42**

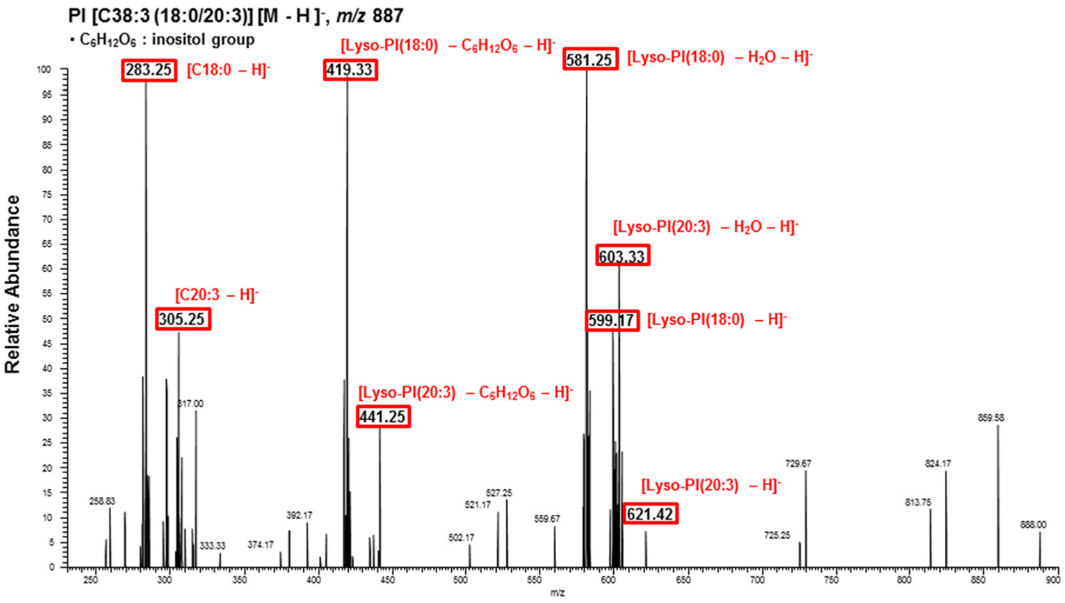


(*Continued*)

**Compound #43**

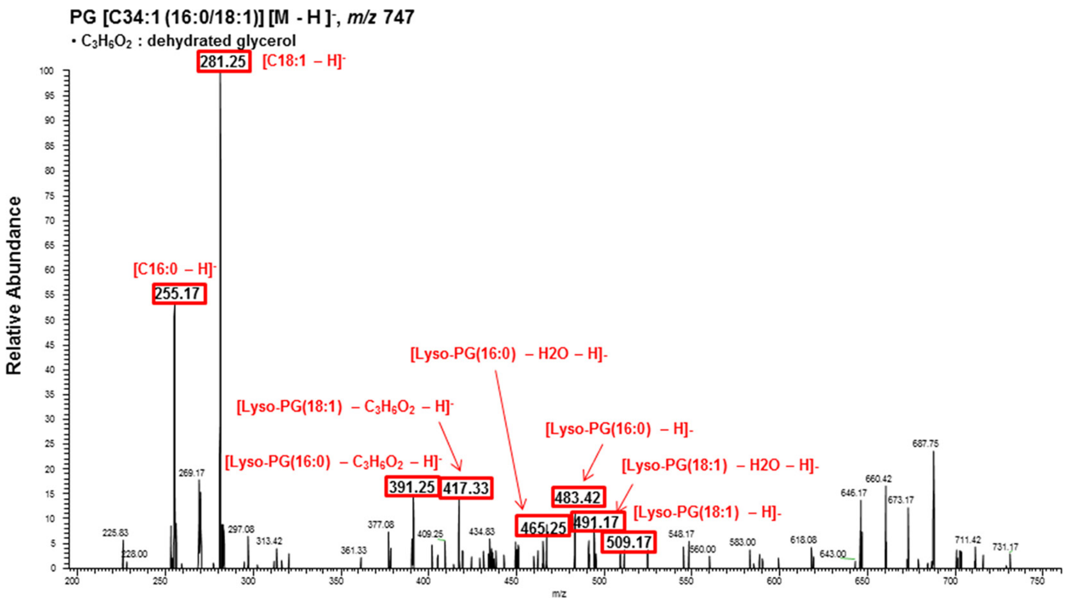


**Compound #44**

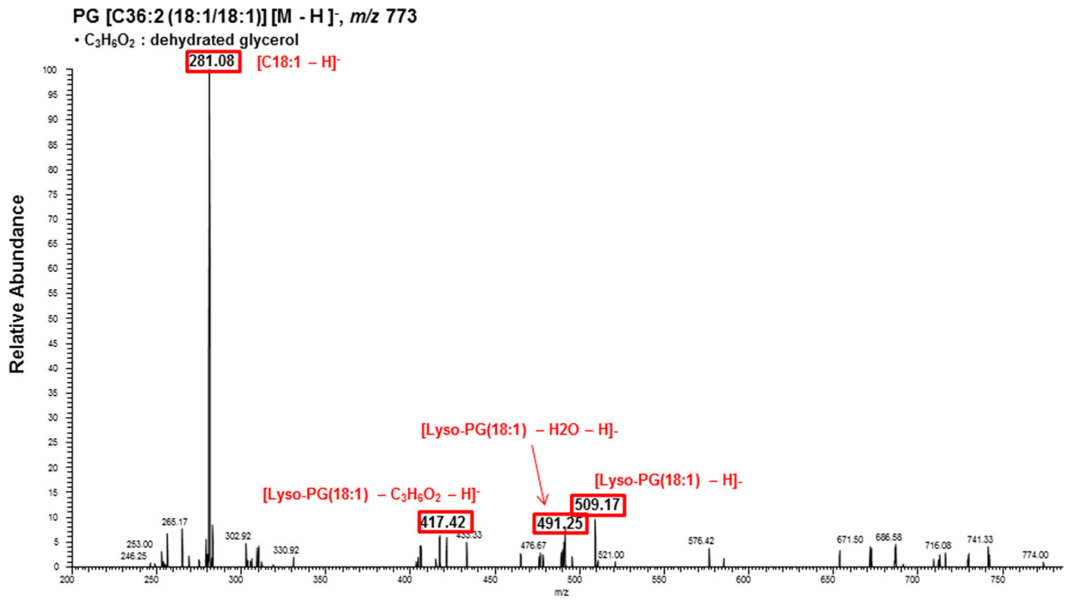


**Supplementary Figure S2: MS/MS spectra of the assigned 44 intact lipids in mammary epithelial and breast cancer cells using LTQ-XL mass spectrometry.** All lipids were identified by matching against the in-house MS/MS library, databases of Lipidmaps and LipidBlast, based on their major structural features, and the major precursor and fragment ions were indicated as red characters. Each compound corresponding to compound # was listed in Supplementary Table S2.

**Supplementary Table S1: A GC-MS-based metabolic profile of breast cancer cells in different progression stages**

| No. | Compound | RT | MCF-10A | MCF-7 | MDA-MB-231 |
|---|---|---|---|---|---|
| | **Alcohol** | | | | |
| 1 | Erythritol | 19.03 | 3.94 ± 0.75[a] | 14.55 ± 7.03[b] | 1.50 ± 0.27[a] |
| | | 19.05 | | | |
| 2 | Myo-inositol | 28.95 | 130.11 ± 41.21[a] | 62.12 ± 28.52[a] | 242.86 ± 87.10[b] |
| | **Amino acid** | | | | |
| 3 | Alanine | 7.00 | 110.92 ± 49.66[ab] | 169.45 ± 56.46[a] | 74.36 ± 16.61[b] |
| 4 | Asparagine | 20.85 | 9.08 ± 2.84[a] | 15.92 ± 5.47[b] | 6.15 ± 1.27[a] |
| 5 | Aspartic acid | 15.06 | 119.17 ± 34.00[a] | 85.77 ± 42.08[ab] | 60.90 ± 13.09[b] |
| | | 17.41 | | | |
| | | 17.43 | | | |
| 6 | Glutamine | 19.78 | 343.20 ± 46.94[a] | 1401.87 ± 770.11[b] | 355.54 ± 45.65[a] |
| | | 19.79 | | | |
| | | 19.81 | | | |
| | | 23.11 | | | |
| 7 | Glycine | 7.47 | 221.07 ± 47.07 | 315.14 ± 99.45 | 362.64 ± 138.97 |
| | | 12.02 | | | |
| | | 12.03 | | | |
| 8 | Leucine | 8.30 | 54.91 ± 7.33 | 101.75 ± 42.91 | 68.48 ± 24.12 |
| | | 11.72 | | | |
| 9 | Lysine | 26.08 | 89.80 ± 18.56 | 150.53 ± 65.83 | 91.28 ± 26.41 |
| 10 | Ornithine | 24.00 | 5.09 ± 1.33[a] | 6.96 ± 2.16[a] | 17.80 ± 3.10[b] |
| 11 | Proline | 11.81 | 38.33 ± 11.90[a] | 112.39 ± 50.66[b] | 25.09 ± 8.62[a] |
| 12 | Serine | 10.81 | 43.61 ± 12.39[a] | 191.98 ± 104.09[b] | 38.99 ± 12.56[a] |
| | | 13.45 | | | |
| 13 | Threonine | 11.74 | 39.35 ± 9.52[a] | 258.60 ± 80.39[b] | 33.90 ± 9.90[a] |
| | | 11.75 | | | |
| | | 14.08 | | | |
| | | 14.09 | | | |
| 14 | Tryptophan | 30.92 | 25.12 ± 8.06[ab] | 42.38 ± 15.92[a] | 18.97 ± 5.16[b] |
| 15 | Tyrosine | 26.37 | 137.08 ± 34.37[a] | 389.82 ± 193.48[b] | 101.24 ± 15.73[a] |
| 16 | Valine | 6.71 | 98.82 ± 30.15[a] | 323.69 ± 105.30[b] | 78.92 ± 26.64[a] |
| | | 9.74 | | | |
| | | 9.76 | | | |

(*Continued*)

| No. | Compound | RT | MCF-10A | MCF-7 | MDA-MB-231 |
|---|---|---|---|---|---|
| | **Fatty acid** | | | | |
| 17 | 3-hydroxybutanoic acid | 8.05 | 26.96 ± 6.97[a] | 22.64 ± 11.89[a] | 49.06 ± 12.80[b] |
| 18 | Oleic acid | 31.08 | 1.41 ± 0.71 | 1.68 ± 1.33 | 2.44 ± 0.60 |
| 19 | Palmitic acid | 28.36 | 13.73 ± 2.11[ab] | 12.54 ± 4.31[a] | 20.13 ± 7.57[b] |
| 20 | Stearic acid | 31.49 | 7.84 ± 3.60 | 8.26 ± 2.33 | 11.92 ± 3.58 |
| | **Organic acid** | | | | |
| 21 | Isocitric acid | 24.07 | 2.14 ± 0.76[a] | 12.26 ± 4.53[b] | 8.64 ± 3.01[b] |
| 22 | Lactic acid | 6.04 | 32.16 ± 7.87[a] | 199.70 ± 85.43[b] | 59.49 ± 23.99[a] |
| 23 | Malic acid | 16.67 | 7.74 ± 2.46[a] | 14.68 ± 4.87[b] | 5.10 ± 1.28[a] |
| 24 | Oxalic acid | 7.90 | 248.51 ± 94.00[a] | 388.76 ± 128.54[ab] | 509.14 ± 194.63[b] |
| 25 | Pyruvic acid | 7.63 | 11.16 ± 7.74 | 11.97 ± 4.66 | 15.44 ± 3.16 |
| | **Purine** | | | | |
| 26 | Adenine | 24.95 | 6.71 ± 1.03[a] | 8.28 ± 1.48[a] | 109.12 ± 13.81[b] |
| 27 | Guanine | 29.59 | 0.37 ± 0.06[a] | 116.02 ± 58.59[b] | 137.75 ± 18.65[b] |
| 28 | Hypoxanthine | 23.73 | 244.10 ± 92.38[a] | 167.28 ± 97.68[ab] | 84.17 ± 9.68[b] |
| 29 | Inosine | 36.11 | 11.30 ± 1.94[a] | 22.60 ± 11.00[b] | 2.34 ± 1.21[a] |
| 30 | Uracil | 12.86 | 22.51 ± 3.40[a] | 2.05 ± 0.76[b] | 29.13 ± 6.33[a] |
| 31 | Xanthine | 27.80 | 20.49 ± 6.32[a] | 0.23 ± 0.35[b] | 2.71 ± 0.74[b] |
| | **Sugar** | | | | |
| 32 | 6-phosphogluconic acid | 33.93 | 4.58 ± 1.35 | 3.18 ± 2.67 | 4.61 ± 2.15 |
| 33 | Fructose-6-phosphate | 32.31 | 34.04 ± 13.35[a] | 5.94 ± 4.14[b] | 12.92 ± 3.98[b] |
| 34 | Glucose | 27.15 | 11.88 ± 8.37[ab] | 20.67 ± 12.67[a] | 7.15 ± 1.70[b] |
| 35 | Glucose-6-phosphate | 32.32 | 187.35 ± 36.35[a] | 41.77 ± 23.83[b] | 118.50 ± 30.10[c] |
| | | 32.49 | | | |
| | | 32.74 | | | |
| | | 33.03 | | | |
| | | 33.33 | | | |
| | | 33.50 | | | |
| 36 | Glyceric acid | 12.67 | 0.94 ± 0.37[a] | 2.43 ± 0.99[b] | 0.92 ± 0.62[a] |
| 37 | Mannose-6-phosphate | 35.72 | 59.41 ± 18.13[a] | 17.48 ± 9.79[b] | 11.95 ± 3.52[b] |
| 38 | Sucrose | 36.96 | 6.20 ± 2.29 | 5.85 ± 0.90 | 8.06 ± 3.77 |
| | **Other** | | | | |
| 39 | Creatinine | 18.10 | 3.86 ± 2.02[a] | 14.50 ± 6.90[b] | 3.10 ± 1.48[a] |

Different letters in the same row indicate a significant difference. Mean ± SD value for quintuplicate measurements are shown. The values are multiplied by ‘10e3’ due to significantly small values

**Supplementary Table S2: Normalized ion intensities of identified lipids in mammary epithelial cells (MCF-10A) and metastatic breast cancer cells (MCF-7 and MDA-MB-231) using nano-electrospray mass spectrometry**

| No. | Lipid molecular species (total acyl carbons:total double bonds) | Proposed composition | Ion species | m/z | MCF-10A | MCF-7 | MDA-MB-231 |
|---|---|---|---|---|---|---|---|
| Positive ion mode | | | | | | | |
| Phosphatidylcholine (PC) | | | | | | | |
| 1 | PC 34:2 | C16:1/C18:1 | $[M + H]^+$ | 758 | $1.77 \pm 0.26^a$ | $3.52 \pm 0.39^b$ | $4.37 \pm 1.07^b$ |
| 2 | PC 36:3 | C18:1/C18:2 | $[M + H]^+$ | 784 | $2.05 \pm 0.42^a$ | $4.93 \pm 0.58^{ab}$ | $8.42 \pm 3.58^b$ |
| 3 | PC 36:2 | C18:1/C18:1 | $[M + H]^+$ | 786 | $9.72 \pm 2.33^a$ | $24.65 \pm 2.90^{ab}$ | $39.53 \pm 15.19^b$ |
| 4 | PC 37:3 | C18:3/C19:0 | $[M + H]^+$ | 798 | 7.45 ± 5.28a | 10.67 ± 3.46a | 8.63 ± 0.97a |
| 5 | PC 38:5 | C18:2/C20:3 | $[M + H]^+$ | 808 | $0.37 \pm 0.12^a$ | $1.19 \pm 0.32^b$ | $1.68 \pm 0.75^b$ |
| 6 | PC 38:4 | C18:0/C20:4 | $[M + H]^+$ | 810 | $0.39 \pm 0.05^a$ | $0.63 \pm 0.12^a$ | $1.76 \pm 0.27^b$ |
| 7 | PC 38:2 | C18:1/C20:1 | $[M + H]^+$ | 814 | $1.36 \pm 0.22^a$ | $2.54 \pm 0.29^b$ | $1.39 \pm 0.23^a$ |
| 8 | PC 38:1 | C18:1/C20:0 | $[M + H]^+$ | 816 | $2.88 \pm 0.49^a$ | $4.52 \pm 0.60^b$ | $5.27 \pm 0.83^b$ |
| 9 | PC 38:0 | C18:0/C20:0 | $[M + H]^+$ | 818 | $3.39 \pm 1.63^a$ | $5.11 \pm 2.16^{ab}$ | $7.73 \pm 2.77^b$ |
| 10 | PC 40:6 | C18:1/C22:5 | $[M + H]^+$ | 834 | $1.33 \pm 0.17^a$ | $2.10 \pm 0.19^b$ | $2.03 \pm 0.21^b$ |
| Plasmenylphosphatidylcholine (plasmenyl-PC) | | | | | | | |
| 11 | Plasmenyl-PC 38:1 | C20:0/C18:1 | $[M + H]^+$ | 800 | $3.87 \pm 1.43^a$ | $7.46 \pm 0.64^b$ | $3.67 \pm 2.00^a$ |
| Plasmenylphosphatidylethanolamine (plasmenyl-PE) | | | | | | | |
| 12 | plasmenyl-PE 34:0 | C18:0/C16:0 | $[M + H]^+$ | 704 | $1.33 \pm 0.49^a$ | $1.98 \pm 0.64^a$ | $2.02 \pm 0.32^a$ |
| Sphingomyelin (SM) | | | | | | | |
| 13 | SM 34:1 | D18:1/16:0 | $[M]^+$ | 703 | $2.07 \pm 0.94^a$ | $2.26 \pm 0.92^a$ | $3.10 \pm 0.76^a$ |
| Negative ion mode | | | | | | | |
| Phosphatidylethanolamine (PE) | | | | | | | |
| 14 | PE 36:2 | C18:1/C18:1 | $[M - H]^-$ | 742 | $10.01 \pm 0.69^a$ | $16.17 \pm 0.67^b$ | $4.68 \pm 0.30^c$ |
| 15 | PE 36:0 | C17:0/C19:0 | $[M - H]^-$ | 746 | $2.79 \pm 0.57^a$ | $7.30 \pm 0.37^b$ | $4.35 \pm 1.37^c$ |
| 16 | PE 38:5 | C18:1/C20:4 | $[M - H]^-$ | 764 | $3.31 \pm 0.27^a$ | $8.87 \pm 0.17^b$ | $4.09 \pm 0.62^c$ |
| 17 | PE 40:5 | C18:1/C22:4 | $[M - H]^-$ | 792 | $4.86 \pm 0.36^a$ | $8.11 \pm 0.47^b$ | $7.51 \pm 1.27^b$ |
| Plasmenylphosphatidylethanolamine (plasmenyl-PE) | | | | | | | |
| 18 | plasmenyl-PE 38:4 | C18:0/C20:4 | $[M - H]^-$ | 750 | $4.55 \pm 0.28^a$ | $1.65 \pm 0.18^b$ | $13.03 \pm 1.02^c$ |
| Phosphatidylserine (PS) | | | | | | | |
| 19 | PS 34:2 | C16:1/C18:1 | $[M - H]^-$ | 758 | $7.56 \pm 0.72^a$ | $15.01 \pm 1.36^b$ | $25.11 \pm 4.40^c$ |
| 20 | PS 34:1 | C16:0/C18:1 | $[M - H]^-$ | 760 | $33.19 \pm 4.34^a$ | $48.96 \pm 3.55^b$ | $80.84 \pm 12.65^c$ |

(*Continued*)

| No. | Lipid molecular species (total acyl carbons:total double bonds) | Proposed composition | Ion species | m/z | MCF-10A | MCF-7 | MDA-MB-231 |
|---|---|---|---|---|---|---|---|
| 21 | PS 34:0 | C16:0/C18:0 | $[M-H]^-$ | 762 | 9.76 ± 0.96$^{a}$ | 11.87 ± 0.82$^{a}$ | 20.15 ± 2.89$^{b}$ |
| 22 | PS 35:1 | C17:0/C18:1 | $[M-H]^-$ | 774 | 5.22 ± 0.56$^{a}$ | 12.82 ± 1.34$^{b}$ | 6.31 ± 0.69$^{a}$ |
| 23 | PS 36:3 | C18:1/C18:2 | $[M-H]^-$ | 784 | 10.54 ± 1.10$^{a}$ | 6.36 ± 0.63$^{a}$ | 34.69 ± 7.56$^{b}$ |
| 24 | PS 36:2 | C18:0/C18:2 | $[M-H]^-$ | 786 | 36.71 ± 2.26$^{a}$ | 36.22 ± 3.41$^{a}$ | 77.96 ± 12.92$^{b}$ |
| 25 | PS 36:1 | C18:0/C18:1 | $[M-H]^-$ | 788 | 51.25 ± 3.70$^{a}$ | 44.11 ± 2.35$^{a}$ | 104.38 ± 12.65$^{b}$ |
| 26 | PS 36:0 | C18:0/C18:0 | $[M-H]^-$ | 790 | 11.46 ± 0.72$^{a}$ | 23.03 ± 0.96$^{b}$ | 24.21 ± 1.86$^{b}$ |
| 27 | PS 38:4 | C18:0/C20:4 | $[M-H]^-$ | 810 | 2.70 ± 0.50$^{a}$ | 2.88 ± 0.18$^{a}$ | 11.97 ± 1.30$^{b}$ |
| 28 | PS 38:1 | C18:1/C20:0 | $[M-H]^-$ | 816 | 7.29 ± 0.42$^{a}$ | 11.43 ± 0.52$^{b}$ | 10.23 ± 0.88$^{c}$ |
| 29 | PS 40:6 | C18:0/C22:6 | $[M-H]^-$ | 834 | 2.91 ± 0.33$^{a}$ | 5.63 ± 0.55$^{b}$ | 12.13 ± 1.18$^{c}$ |
| 30 | PS 40:5 | C18:1/C22:4 | $[M-H]^-$ | 836 | 5.35 ± 0.60$^{a}$ | 13.14 ± 0.75$^{b}$ | 12.88 ± 1.32$^{b}$ |
| 31 | PS 40:4 | C18:0/C22:4 | $[M-H]^-$ | 838 | 2.82 ± 0.32$^{a}$ | 3.01 ± 0.26$^{a}$ | 10.62 ± 1.61$^{b}$ |
| 32 | PS 40:2 | C18:1/C22:1 | $[M-H]^-$ | 842 | 5.38 ± 0.26$^{a}$ | 3.46 ± 0.06$^{b}$ | 5.76 ± 0.52$^{a}$ |
| 33 | PS 40:1 | C18:1/C22:0 | $[M-H]^-$ | 844 | 9.54 ± 0.58$^{a}$ | 15.09 ± 0.72$^{b}$ | 13.10 ± 1.15$^{c}$ |
| Phosphatidylinositol (PI) | | | | | | | |
| 34 | PI 32:1 | C16:0/C16:1 | $[M-H]^-$ | 807 | 2.22 ± 0.15$^{a}$ | 7.17 ± 0.29$^{a}$ | 37.76 ± 8.27$^{b}$ |
| 35 | PI 34:2 | C16:1/C18:1 | $[M-H]^-$ | 833 | 5.50 ± 0.44$^{a}$ | 6.28 ± 0.43$^{a}$ | 11.56 ± 1.42$^{b}$ |
| 36 | PI 34:1 | C16:0/C18:1 | $[M-H]^-$ | 835 | 9.49 ± 0.76$^{a}$ | 23.30 ± 1.44$^{b}$ | 13.32 ± 2.38$^{c}$ |
| 37 | PI 36:2 | C18:1/C18:1 | $[M-H]^-$ | 861 | 27.51 ± 1.95$^{a}$ | 18.17 ± 1.25$^{b}$ | 6.75 ± 0.60$^{c}$ |
| 38 | PI 36:1 | C18:0/C18:1 | $[M-H]^-$ | 863 | 21.06 ± 1.47$^{a}$ | 35.18 ± 2.23$^{b}$ | 3.39 ± 0.41$^{c}$ |
| 39 | PI 38:6 | C16:0/C22:6 | $[M-H]^-$ | 881 | 2.02 ± 1.11$^{a}$ | 3.19 ± 0.36$^{a}$ | 7.73 ± 3.08$^{b}$ |
| 40 | PI 38:5 | C18:1/C20:4 | $[M-H]^-$ | 883 | 1.63 ± 0.27$^{a}$ | 2.04 ± 0.19$^{a}$ | 6.89 ± 1.28$^{b}$ |
| 41 | PI 38:4 | C18:0/C20:4 | $[M-H]^-$ | 885 | 10.73 ± 0.86$^{a}$ | 7.42 ± 0.57$^{a}$ | 62.18 ± 7.40$^{b}$ |
| 42 | PI 38:3 | C18:0/C20:3 | $[M-H]^-$ | 887 | 11.90 ± 0.82$^{a}$ | 5.74 ± 0.49$^{b}$ | 13.90 ± 1.61$^{c}$ |
| Phosphatidylglycerol (PG) | | | | | | | |
| 43 | PG 34:1 | C16:0/C18:1 | $[M-H]^-$ | 747 | 4.99 ± 0.61$^{a}$ | 8.17 ± 0.61$^{b}$ | 3.61 ± 0.95$^{c}$ |
| 44 | PG 36:2 | C18:1/C18:1 | $[M-H]^-$ | 773 | 4.40 ± 0.61$^{a}$ | 8.90 ± 1.34$^{b}$ | 0.96 ± 0.27$^{c}$ |

[a] Each value represents the mean ± standard deviation (SD) (n = 5).

[b] For simplicity, '×10e1' and '×10e2' were omitted from the values of identified lipids in positive mode and negative mode, respectively.
